# KSHV 2.0: A Comprehensive Annotation of the Kaposi's Sarcoma-Associated Herpesvirus Genome Using Next-Generation Sequencing Reveals Novel Genomic and Functional Features

**DOI:** 10.1371/journal.ppat.1003847

**Published:** 2014-01-16

**Authors:** Carolina Arias, Ben Weisburd, Noam Stern-Ginossar, Alexandre Mercier, Alexis S. Madrid, Priya Bellare, Meghan Holdorf, Jonathan S. Weissman, Don Ganem

**Affiliations:** 1 Novartis Institute for Biomedical Research, Department of Infectious Diseases, Emeryville, California, United States of America; 2 Novartis Vaccines and Diagnostics, Bioinformatics, Emeryville, California, United States of America; 3 Department of Cellular and Molecular Pharmacology, Howard Hughes Medical Institute, University of California, San Francisco, San Francisco, California, United States of America; University of North Carolina at Chapel Hill, United States of America

## Abstract

Productive herpesvirus infection requires a profound, time-controlled remodeling of the viral transcriptome and proteome. To gain insights into the genomic architecture and gene expression control in Kaposi's sarcoma-associated herpesvirus (KSHV), we performed a systematic genome-wide survey of viral transcriptional and translational activity throughout the lytic cycle. Using mRNA-sequencing and ribosome profiling, we found that transcripts encoding lytic genes are promptly bound by ribosomes upon lytic reactivation, suggesting their regulation is mainly transcriptional. Our approach also uncovered new genomic features such as ribosome occupancy of viral non-coding RNAs, numerous upstream and small open reading frames (ORFs), and unusual strategies to expand the virus coding repertoire that include alternative splicing, dynamic viral mRNA editing, and the use of alternative translation initiation codons. Furthermore, we provide a refined and expanded annotation of transcription start sites, polyadenylation sites, splice junctions, and initiation/termination codons of known and new viral features in the KSHV genomic space which we have termed KSHV 2.0. Our results represent a comprehensive genome-scale image of gene regulation during lytic KSHV infection that substantially expands our understanding of the genomic architecture and coding capacity of the virus.

## Author Summary

Kaposi's sarcoma-associated herpesvirus (KSHV) is a cancer-causing agent in immunocompromised patients that establishes long-lasting infections in its hosts. Initially described in 1994 and extensively studied ever since, KSHV molecular biology is understood in broad outline, but many detailed questions are still to be resolved. After almost two decades, specific aspects pertaining to the organization of the KSHV genome as well as the fate of the viral transcripts during the productive stages of infection remain unexplored. Here we use a systematic genome-wide approach to investigate changes in gene and protein expression during the productive stage of infection known as the lytic cycle. We found that the viral genome has a large coding capacity, capable of generating at least 45% more products than initially anticipated by bioinformatic analyses alone, and that it uses multiple strategies to expand its coding capacity well beyond what is determined solely by the DNA sequence of its genome. We also provide an expanded and highly detailed annotation of known and new genomic features in KSHV. We have termed this new architectural and functional annotation KSHV 2.0. Our results indicate that viral genomes are more complex than anticipated, and that they are subject to tight mechanisms of regulation to ensure correct gene expression.

## Introduction

Kaposi's sarcoma-associated herpesvirus (KSHV) is a member of the gamma-herpesvirus family and the etiologic agent of Kaposi's sarcoma, primary effusion lymphoma (PEL), and multicentric Castleman's disease [1], [2]. This human pathogen, initially identified in Kaposi's sarcoma lesions from AIDS patients, has been extensively studied since its discovery and isolation in 1994 [3]. Shortly thereafter, the KSHV genome, a dsDNA molecule of ∼165 kb, was sequenced from the lymphoid cell line BC-1, allowing the *in silico* annotation of open reading frames (ORFs) that fit the following criteria: (1) they start with a canonical initiator AUG codon and (2) they encode polypeptides larger than 100 amino acids (aa). Many of these ORFs had functional homologues in herpesvirus saimiri (HVS), a gamma-herpesvirus related to KSHV [4]. This study identified a total of 81 such viral ORFs, and except for the more recent addition of microRNAs, non-coding RNAs, and a few small ORFs [5]–[8], the genome map of KSHV has changed little ever since. Gene expression profiling of KSHV transcripts using northern blots, custom oligonucleotide microarrays and real time PCR arrays have demonstrated extensive transcription of the viral genome, hinting at a complex transcriptional profile [6], [9], [10] (unpublished data). More recently, proteomic studies of KSHV-infected cells have assessed the expression of many of the predicted ORFs [11]. However, and in spite of all the aforementioned efforts, a detailed understanding of the genomic architecture, translational state, and biological functions of KSHV gene products remains incomplete.

In an attempt to extend our current knowledge of the coding capacity of the KSHV genome during the productive stage of infection, we employed an unbiased functional genomics approach to study the transcription and translation profiles of lytic KSHV using mRNA-sequencing (mRNA-Seq), ribosome footprinting (Ribo-Seq), and genomic DNA sequencing (DNA-Seq). When combined, these methods provide a comprehensive, high-resolution view of gene regulation and expression dynamics [12]–[14].

By employing these techniques in parallel, we have generated a state-of-the-art annotation of the KSHV genome. Our approach confirms the presence and timing of expression of the majority of previously annotated ORFs, while revealing several novel and, in some cases, unexpected genomic features including ribosome protection of non-coding RNAs, new splice variants, and a plethora of upstream and small ORFs. In addition, we have confirmed and expanded the annotation of transcription start sites, polyadenylation sites, and initiation/termination codons of multiple known ORFs. Our analyses have also uncovered new instances of viral mRNA editing, strongly hinting at a new layer of viral gene regulation during reactivation. The wealth of information generated by integrating the data obtained from our combined methods has expanded our understanding of the viral genome architecture and dynamics, revealing a surprising coding capacity of KSHV that goes well beyond what was initially described based on its genome sequence alone.

## Results

### mRNA-Seq and Ribo-Seq reveal the architecture of the coding and non-coding viral transcriptome at a single-nucleotide resolution

The life cycle of KSHV can be separated in two very distinct stages: the dormant state known as latency and the productive state referred to as the lytic cycle [2]. While viral gene expression in latency is limited and most of the genome is silent, the lytic cycle is a transcriptionally dynamic state where the timing of gene expression is tightly regulated to ensure the ordered synthesis of viral products [6], [15], [16]. We sought to study the kinetics of latent and lytic viral transcription in detail, as well as the translational fate of newly synthesized mRNAs. To this end, we employed a system developed by our group that allows the study of the KSHV lytic cycle in a tightly-controlled manner [17]. This system comprises the epithelial iSLK-219 cell line, which is latently infected with a heterologous KSHV strain (see below) and harbors a doxycycline (Dox) inducible transgene encoding the viral transcription factor RTA (replication and transcriptional activator). The exogenous expression of RTA by Dox treatment in iSLK-219 cells is sufficient to induce the lytic reactivation of latent KSHV. Notably, latency in iSLK-219 cells is very strict with less than 0.1% of the cells showing lytic markers in the absence of induction [17]. This is the principal experimental advantage of SLK cells, allowing the study of KSHV latency in the near total absence of contaminating lytically infected cells. The viral strain in iSLK-219 is the recombinant KSHV.219, which encodes a constitutive GFP reporter as well as an RTA-inducible RFP reporter in the viral genome, thereby facilitating the monitoring of viral reactivation [18].

**Figure 1:**
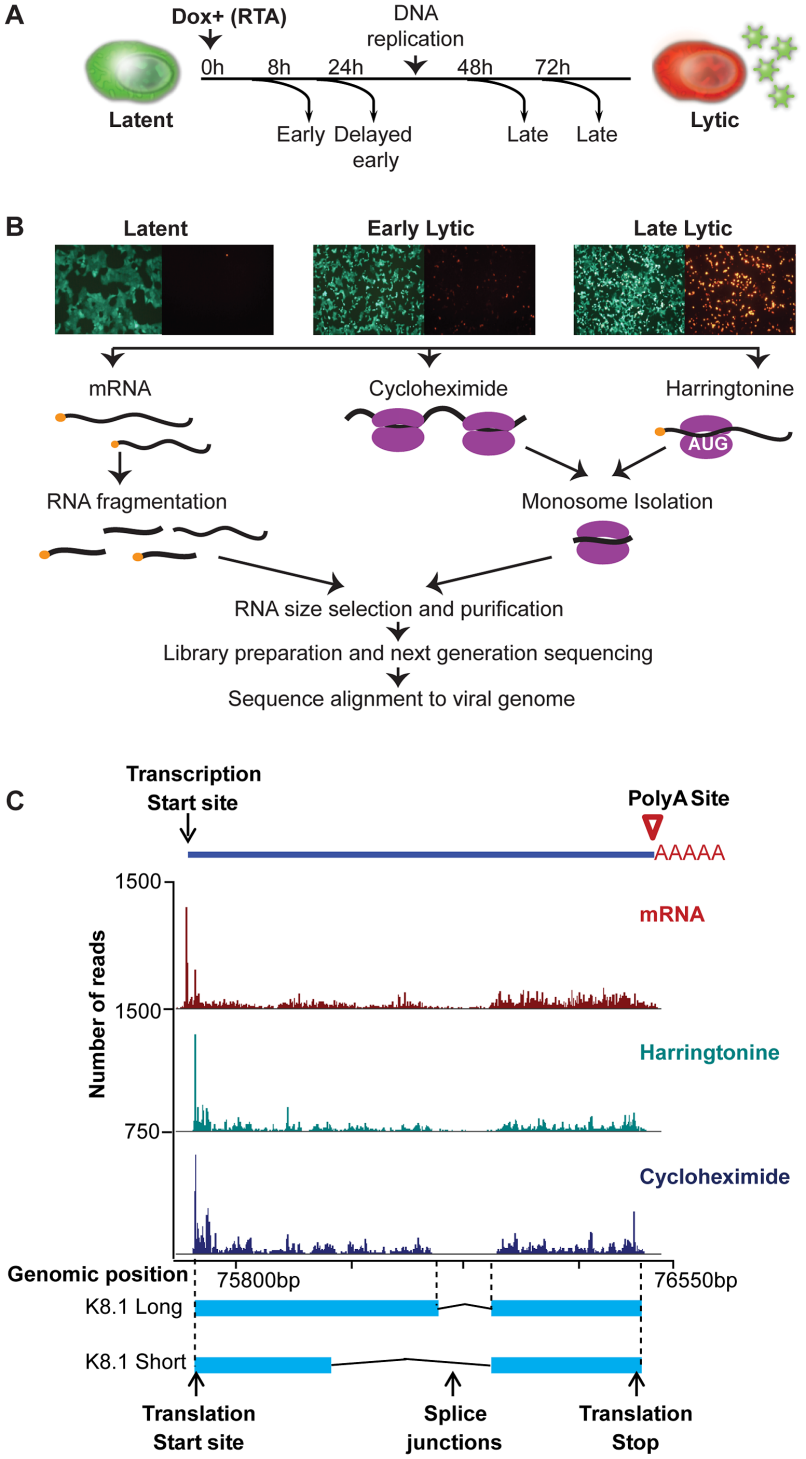
High-resolution mapping of KSHV genomic features. (A–B) Strategy for identification of transcripts and ribosome footprints in iSLK-219 cells. Latent iSLK-219 cells were induced to enter the lytic cycle by expression of the doxycycline-inducible transcription factor RTA for 8, 24, 48 and 72 hr. mRNA and ribosome footprints were isolated from cells and the purified RNA was deep sequenced. (C) Ribosome occupancy and mRNA profiles of the late lytic gene K8.1 at 48 hr post induction (hpi).

To finely resolve the transcriptional profile and the ribosome occupancy of viral mRNAs, we induced iSLK-219 cells with Dox for 0, 8, 24, 48 and 72 hr (Figure 1A). We evaluated KSHV lytic reactivation by epifluorescence microscopy analysis of GFP and RFP expression, as well as by immunodetection of viral products and quantification of viral DNA replication (Figure 1B, Figure S1). In iSLK-219, lytic DNA replication, the traditional border between early and late times, commences at ∼48 hr post induction (hpi) (Figure S1B). The selected time points represent the different stages of the lytic cycle, known as latent (0 hr), immediate early- (8 hr), delayed early- (24 hr), and late-lytic (48 and 72 hr) (Figure 1A). At each time point, we recovered polyadenylated RNA (mRNA) and 3 sets of ribosome footprints (described in Materials and Methods). To map actively elongating ribosomes on viral transcripts, we isolated ribosome footprints from cells treated with cycloheximide (CHX), a translation inhibitor that binds the ribosomal-E-site and arrests elongating ribosomes [19]. In the same manner, we mapped initiating ribosomes by treating cells with harringtonine (Harr), a translational inhibitor that binds the 60S subunit and hinders the progression of the initiating ribosome, causing ribosomes to stall at translation start sites [20]. Finally we mapped releasing ribosomes accumulating at the stop codon in samples that were not treated with any drug as previously described [14]. We then constructed Illumina-compatible libraries from fragmented and size-selected mRNA segments (40–100 nt), or ribosome protected RNA (ribosome footprints ∼30 nt in length, Figure S2A) following the standard ribosome profiling protocol previously described [14], [21]. The libraries were deep-sequenced and the resulting reads aligned to the KSHV genome (GQ994935). As expected, the number of reads aligning to the KSHV genome increased as the lytic cycle progressed (Figure S2B). To annotate viral splice junctions, we used two splice junction mapping tools; TopHat and HMMSplicer [22], [23]. With these tools, we detected the majority of the known splice junctions and discovered 7 new events including one at the 3′ end of ORF57. Lastly, we annotated putative ORFs by training a support vector machine (SVM) to identify translation initiation sites throughout the KSHV genome based on characteristic peaks within the harringtonine Ribo-Seq data. The list of ORFs produced by extending each of the putative initiation sites to the next in-frame stop codon (taking into account any intervening splice junctions) was then finalized through manual curation.

Figure 1C shows an example of the data obtained with our combined approach. In this case the read coverage from mRNA-Seq and Ribo-Seq (CHX and Harr) libraries for the late-lytic viral gene K8.1, one of the best-mapped genes in KSHV. The data clearly illustrates the single-nucleotide resolution and high-coverage of our methods which here allow the delineation of transcription start and end sites, splice junctions, and coding region boundaries. Notably, the coordinates derived from our combined approach correspond precisely to those previously reported for this gene [24], [25], providing strong validation of our methodology. Together, the data obtained using our multipronged approach generated a high-resolution map of the viral genome architecture.

### KSHV 2.0: A revised, high-resolution functional and temporal annotation of the KSHV genome

**Figure 2:**
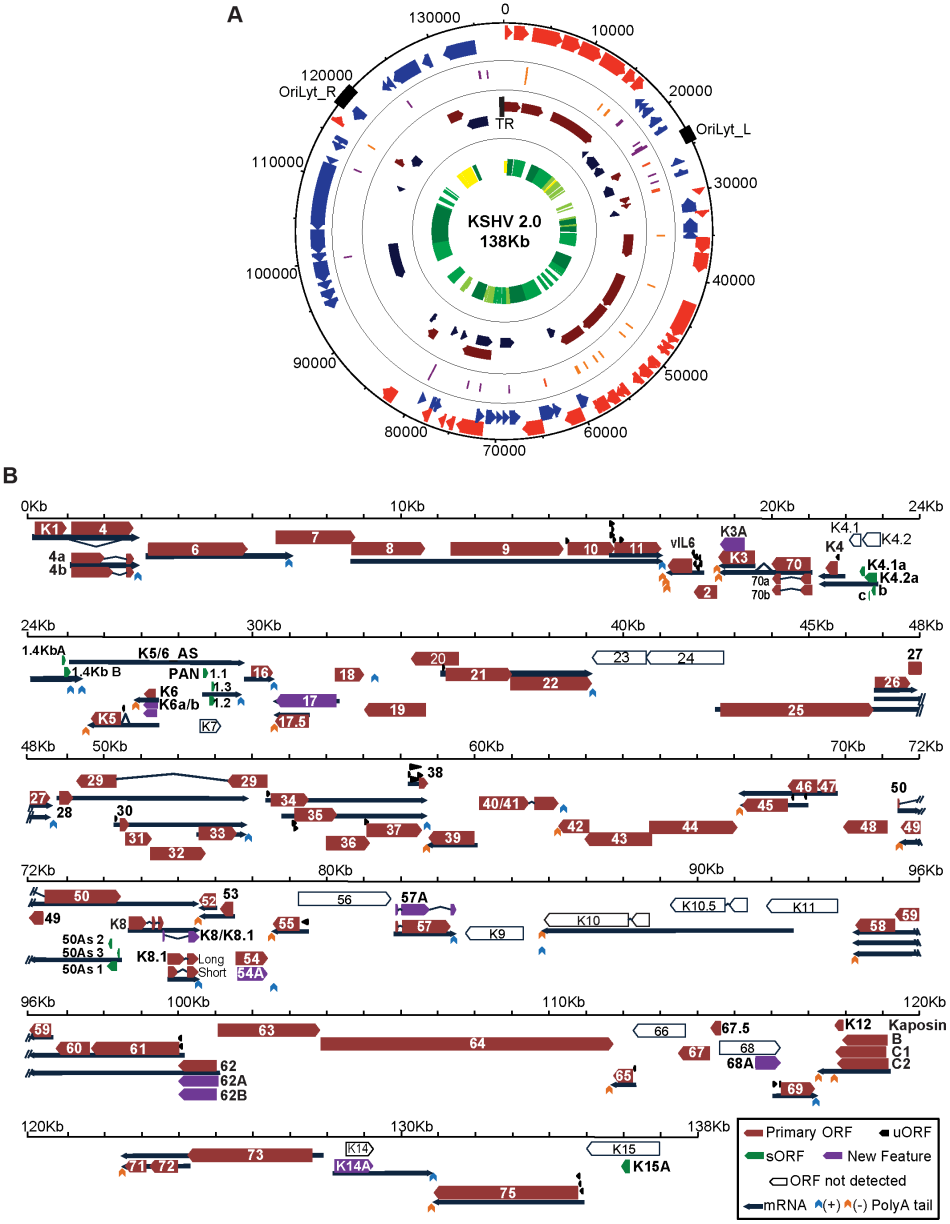
KSHV 2.0: A high-resolution functional genome map of KSHV. (A) Circular map of the KSHV genome. The outer circle represents the genomic coordinates. The concentric circles illustrate, from the outermost to the innermost: viral ORFs (Red sense, blue antisense), sORFs (purple) and uORFs (orange), transcripts (maroon sense, dark blue antisense) and timing of expression (from yellow to dark green: latent, early lytic, delayed early lytic, late lytic). (B) Linear map of the KSHV genome showing the details of genomic features illustrated in A. ORF29, ORF32 and ORF36 are depicted as annotated ORFs as we detected the presence of ribosomes in their coding sequences. The exact boundaries of translation for these ORFs could not be determined. See also Tables 1, 2 and 3.

mRNA-Seq and Ribo-Seq allowed us to perform an unabridged temporal analysis of viral gene expression coupled to a blueprint of the viral episome architecture, granting the opportunity to develop a revised version of the KSHV genome annotation, which we have designated KSHV 2.0 (Figure 2 and Tables 1, 2 and 3). In KSHV 2.0 we annotate the coordinates for 49 viral transcripts and 70 ORFs, as well as those for non-coding RNAs, polyadenylation signals, and splice junctions. In addition, KSHV 2.0 incorporates information pertaining to the timing of expression of the aforementioned elements. Remarkable novel features of KSHV 2.0 include a set of 50 ribosome-loaded segments not previously annotated as bona fide ORFs because of their small size (3–100 aa) and the use of non-canonical start sites (Figure 2B). Together with novel peptide isoforms, and splice variants, these short and upstream ORFs (sORFs and uORFs, respectively) increase the coding repertoire of KSHV by more than 45% and add a new level of potential gene regulation to an already complex landscape. The novel features annotated in this study are summarized in file S1 and can be visualized using the mochiview database in file S2 [26]. In spite of the comprehensive annotation generated for KSHV 2.0, some known features of the viral genome were not detected or could not be rigorously assigned (Figure 2B), due to ambiguities generated by regions of low sequencing coverage, overlapping transcription and translation, and cell line specific patterns of gene expression.

**Table 1:** Transcription and translation coordinates, timing of expression, and function of latent, early lytic and delayed early lytic ORFs.

Gene	Str	TSS-PolyA	Start-Stop	Ann	Variant	Timing	Function	Ref
ORFK1	+	38–2956	105–959	P		Latent	Glycoprotein	[89]
ORF4	+	1097–2956	1127–2779	P	Sp.Var	24 h	Complement binding protein	[90]
ORF6^U^	+	3137–7013	3194–6592	P		24–48 h	ssDNA Binding protein	[91]
ORF11^U^	+	15610–17054	15771–16994	P		8 h	Predicted dUTPase	[92]
vIL6**^U^**	−	17960–17163	17856–17242	P		Latent	viral Interleukine 6 homolog	[93]
ORFK3	−	21099–18575	19557–18589	P	Internal	24 h	Immune modulator	[94], [95]
ORF70	−	21099–18575	21051–20038	P	Alt. Start	8 h	thymidylate synthasee	[96]
ORFK4	−	21898–21274	21779–21495	P		8 h	vMIP-II	[97]
ORFK4.1	−	22864–21274	N/A	P		8 h	N/A	
1.4 kb	+	24175–25394	multiple	P		8 h	N/A	[28]
ORFK5^U^	−	26692–25501	26433–25663	P		8 h	RING-CH E3 Ubiquitin ligase	[94], [95]
ORFK6	−	27472–26843	27374–27422	P	Alt. Start	8 h	vMIP-IA	[94], [95]
PAN	+	28616–29690	multiple	P		8 h	Late gene expression	[33], [38]
ORF16	+	29770–30566	29962–30489	P		8 h	Bcl2 homolog	[98], [99]
ORF17.5	−	31536–30563	31505–30639	P		24 h	Assembly protein	[100]
ORF18	+	N/A-33273	32242–33015	P		24 h	Late gene regulation^MHV68^	[101]
ORF34^U^	+	54379–58691	54491–55474	M		24 h–48 h	N/A	[102]
ORF35^U^	+	55381–58691	55455–55907	M		24 h–48 h	N/A	[103]
ORF36	+	N/A-58691	N/A-57126	M		24 h–48 h	Serine protein kinase	[104]
ORF37^U^	+	N/A-58691	57089–58549	M		24 h–48 h	Sox	[50]
ORF38^U^	+	58186–58691	58504–58689	P		24 h–48 h	Myristylated protein	[61]
ORF39	−	60050–58701	59991–58789	P		24 h–48 h	Glycoprotein M	[105]
ORF45^U^	−	68683–67140	68392–67169	P		8 h	RSK activator	[106]
ORF46	−	69782–67140	69220–68453	P		24 h	Uracil deglycosylase	[107]
ORF47^U^	−	69782–67140	69731–69228	M		24 h	glycoprotein L	[96], [108]
ORF50	+	71374–76554	71412–74445	P	AS-ORF	8 h	RTA	[109]
ORFK8	+	74660–76554	74675–75607	P	Sp. Var	8 h–24 h	bZIP	[110]
ORF57	+	81819–83453	81886–83361	P	Sp. Var	8 h	mRNA export/splicing	[61]
ORF58	−	96613–94287	95363–94290	P		24 h	N/A	[61]
ORF59	−	96613–94287	96558–95368	P		24 h	Processivity factor	[61], [62]
ORF60	−	100149–94287	97606–96689	P		24 h–48 h	Ribonucleoprotein reductase	[61]
ORF61^U^	−	100149–94287	100013–97635	P		24 h–48 h	Ribonucleoprotein reductase	[61]
ORFK12	−	119205–117250	119075–117738	P	Alt. Start	Latent	Kaposin	[111]
ORF71	−	124220–122513	123154–122588	M		Latent	vFLIP	[112]
ORF72^U^	−	124220–122513	124010–123236	M		Latent	vCyclin	[112]
ORF73	−	128213–122513	127623–124252	M		Latent	LANA	[112]

Transcription and translation coordinates for the primary viral ORFs were annotated from in silico predictions and/or by manually curating mRNA-seq and Ribo-seq data. Timing was determined according to the time of transcription. Functions were gathered from the literature. U: uORF in leader region; Str: Strand; TSS: transcription start site; N/A, not determined in silico or by manual annotation; Ann: Annotation; P: predicted; M: manual annotation; Sp. Var: ORF with splice variant, Alt. Start: ORF with alternative starts; Internal: ORF with internal ORFs; AS-ORF: Antisense ORFs; MHV68: Function annotated in MHV68.

**Table 2:** Transcription and translation coordinates, timing of expression, and function of late lytic ORFs.

Gene	Str	TSS-PolyA	Start-Stop	Ann	Variant	Timing	Function	Ref
ORF8	+	8656–17054	8680–11217	P		48 h–72 h	Glycoprotein B	[113]
ORF9	+	N/A-17054	11344–14382	P		48 h–72 h	DNA Polymerase	[114]
ORF10^U^	+	N/A-17054	14500–15756	M		48 h–72 h	Regulator of Interferon Function	[115]
ORFK3A	−	21099–18575	19128–18589	P		48 h	Immune modulator	[94], [95]
K5/6-AS	+	N/A-29690	N/A	M		48 h	N/A	[28]
ORF17	−	32300–30563	32243–30639	P		48 h	Protease	[116]
ORF21^U^	+	35120–39147	35201–36943	M		48 h–72 h	Thymidine kinase	[117]
ORF22	+	N/A-39147	36931–39123	P		48 h–72 h	Glycoprotein H	[108]
ORF23	−	N/A-39048	N/A	N/A		48 h–72 h	Glycoprotein (predicted)	[118]
ORF24	−	N/A-39048	N/A	N/A		48 h–72 h	Essential for replication^MHV68^	[119]
ORF25	+	42378–48596	42595–46725	P		48 h–72 h	Major capsid protein	[120]
ORF26	+	46724–48596	46751–47668	P		48 h–72 h	Minor capsid protein	[120]
ORF27	+	N/A-48596	47690–48562	P		48 h–72 h	Glycoprotein^MHV68^	[121]
ORF28^U^	+	48748–53916	48808–49116	P		48 h–72 h	BDLF3 EBV homolog	[122]
ORF29	−	N/A	N/A	N/A		72 h	packaging protein	[123]
ORF30^U^	+	N/A-53916	50440–50673	M		48 h–72 h	Late gene regulation^MHV68^	[124]
ORF31	+	N/A-53916	50580–51254	M		48 h–72 h	Nuclear and cytoplasmic^MHV68^	[124]
ORF32	+	N/A-53916	N/A-52585	M	Internal	48 h–72 h	Tegument protein	[124]
ORF33	+	52553–53916	52578–53582	P		48 h–72 h	Tegument protein^MHV68^	[125]
ORF40/41	+	N/A-62375	60124–62260	P		48 h–72 h	Helicase-Primase	[126]
ORF42	−	63108–62232	63088–62252	P		48 h–72 h	Tegument protein	[127]
ORF43	−	N/A-62232	64769–62952	M	AS-ORF	48 h–72 h	Portal protein (capsid)	[128]
ORF44	+	N/A-67134	64708–67074	M		48 h–72 h	Helicase	[114]
ORF45.1		68952–67140	multiple	P		48 h	N/A	
ORFK8.1	+	75716–76554	75731–76511	P	Sp. Var	48 h	glycoprotein	[24], [25]
ORF52	−	77063–76523	77013–76618	P		48 h–72 h	Tegument protein	[129]
ORF53	−	77513–76523	77481–77149	P		48 h–72 h	Glycoprotein N	[105]
ORF54	+	N/A-78588	77483–78439	M	Alt. Start	48 h–72 h	dUTPase/Immunmodulator	[53]
ORF55**^U^**	−	79525–78521	79264–78581	P		48 h–72 h	Tegument protein	
ORF56	+	N/A-83490	N/A	N/A		48 h–72 h	DNA replication	[61]
ORFK9	−	N/A-83605	N/A	N/A		48 h–72 h	vIRF1	[130]
ORFK10	−	N/A-85823	N/A	N/A		48 h–72 h	vIRF4	[130]
ORFK10.5	−	N/A-89190	N/A	N/A		48 h–72 h	vIRF3	[130]
ORFK11	−	N/A-91573	N/A	N/A		48 h–72 h	vIRF2	[130]
ORF62	−	101148–94287	101013–100018	P	Alt. Start	72 h	N/A	[61]
ORF65^U^	−	112340–111650	112262–111750	P		48 h–72 h	capsid	[131]
ORF66	−	N/A-111650	N/A	N/A		48 h–72 h	capsid	[132]
ORF67	−	N/A-111650	114327–113512	M		48 h–72 h	Nuclear egress complex	[133]
ORF67.5	−	N/A-111650	114624–114382	P		48 h–72 h	N/A	
ORF68	+	N/A-117240	multiple	P	Internal	48 h–72 h	Glycoprotein	[134]
ORF69	+	116029–117240	116257–117165	P		48 h–72 h	BRLF2 Nuclear egress	[133], [134]
ORFK14	+	128171–130870	128210–129256	P		24 h–48 h	vOX2	[135]
ORF74	+	128171–130870	N/A	N/A		24 h–48 h	vGPCR	[135]
ORF75^U^	−	134923–130850	134766–130876	P		48 h–72 h	FGARAT	[136]

Transcription and translation coordinates for the primary viral ORFs were annotated from in silico predictions and/or by manually curating mRNA-seq and Ribo-seq data. Timing was determined according to the time of transcription. Functions were gathered from the literature. U: uORF in leader region; Str: Strand; TSS: transcription start site; N/A, not determined in silico or by manual annotation; Ann: Annotation; P: predicted; M: manual annotation; Sp. Var: ORF with splice variant, Alt. Start: ORF with alternative starts; Internal: ORF with internal ORFs; AS-ORF: Antisense ORFs; MHV68: Function annotated in MHV68.

**Table 3:** Transcription and translation coordinates, and function of primary ORFs without determined time of expression.

Gene	Str	TSS-PolyA	Start-Stop	Ann	Variant	Timing	Function	Ref
ORF2	−	N/A	18534–17902	P		N/A	dihydrofolate reductase	[137], [138]
ORF7	+	N/A	6609–8696	M		N/A	virion protein	[136]
ORF48	−	N/A	71197–69989	P		N/A	N/A	
ORF49	−	N/A-71432	72354–71446	P		N/A	Activates JNK/p38	[139]
ORF63	+	N/A	101027–103813	P		N/A	NLR homolog	[140]
ORF64	+	N/A	103819–111726	P		N/A	Deubiquitinase	[139]
ORFK15	−	N/A	N/A	N/A	Internal	N/A	LMP1/2	[141]

Timing of expression could not be determined for these genes due to overlapping transcripts or low sequencing coverage. Str: Strand; TSS: transcription start site; N/A, not determined in silico or by manual annotation; Ann: Annotation; P: predicted; M: manual annotation; Internal: ORF with internal ORFs.

Inspection of KSHV 2.0 reveals three prominent features: (1) coding and non-coding elements are densely packed in the episome, (2) multiple strategies are used to increase its polypeptide repertoire, including splicing, mRNA editing, and alternative start codon use, and (3) sORFs and uORFs populate many regions of the viral genome. The specific transcriptional and translational features of KSHV 2.0 are discussed in detail below.

### mRNA-Seq reveals a dense viral transcriptome encoded in the KSHV genome

The transcriptional capacity of the KSHV genome has been traditionally studied using northern blotting and gene expression profiling with oligonucleotide microarrays [6], [9], [27], [28]. While these studies have exposed many features of the viral transcriptome, the limitations of these methods prevent the fine mapping of transcripts, which can require single-nucleotide resolution. For this reason, we performed mRNA-Seq in cells lytically infected with KSHV, to explore the transcriptional landscape of the KSHV genome, resolve the boundaries of viral messages, and uncover novel cis-regulatory elements including transcription start sites (TSS), polyadenylation signals (PAS), and splice junctions.

**Figure 3:**
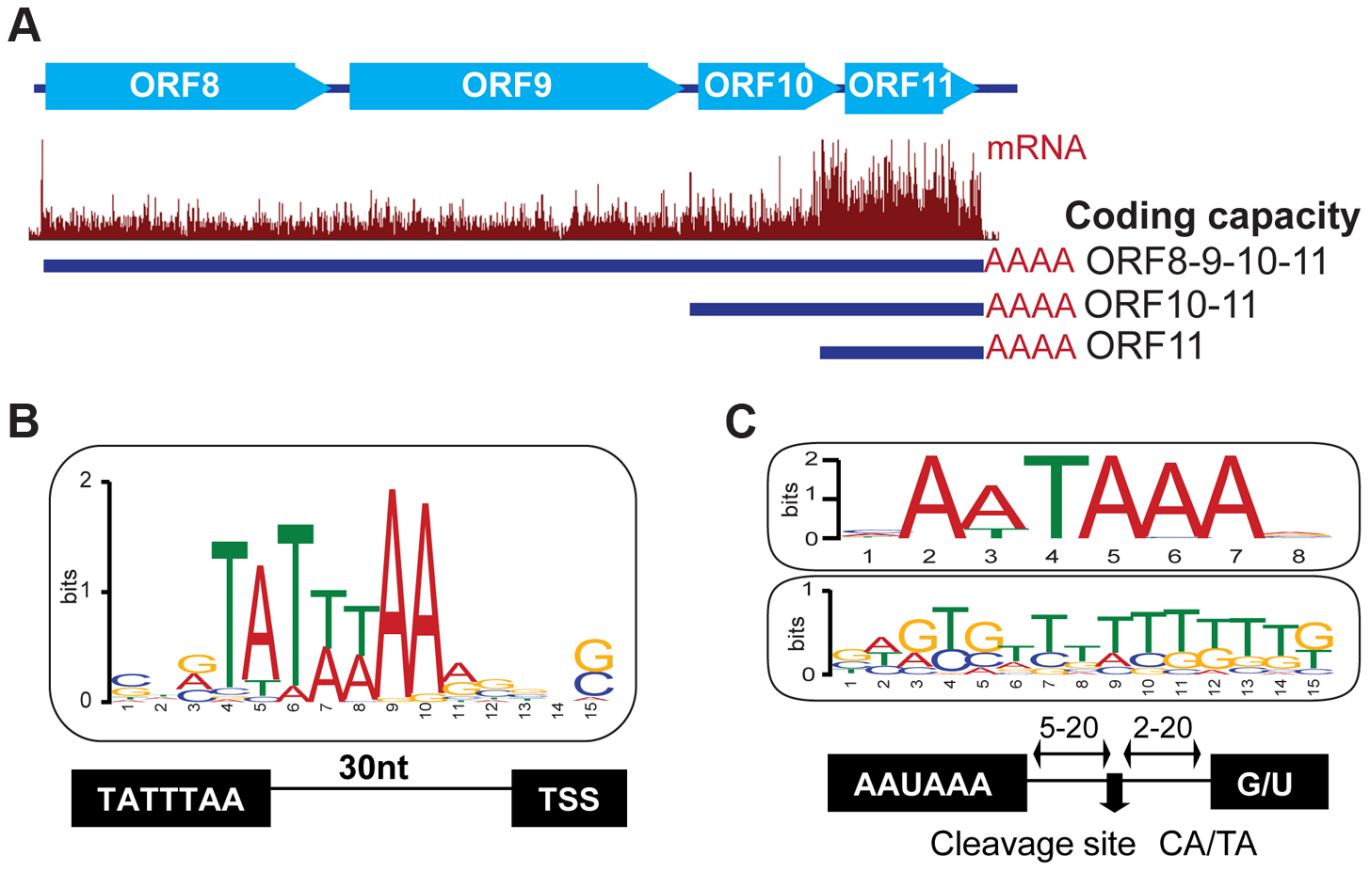
Host cis-regulatory elements are conserved in viral transcripts. (A) mRNA-Seq of the locus encoding the ORF8/9/10 and 11 genes at 72 hr post reactivation. Three TSS sharing a common PAS were mapped within this polycistronic locus. (B) Sequence analysis of 50 nucleotides flanking the predicted TSS site for 49 viral genes. Note the presence of the TATA box 30 bp upstream of the TSS. (C) Sequence analysis of 30 nt flanking the 42 polyA sites mapped in KSHV. Note the conserved AA/UUAAA motif 5–20 nt upstream (top panel), and the GU rich region (middle panel) 5–20 nt downstream of the cleavage site. See also Tables S1 and S2.

Taking advantage of the peaks visible at the 5′ ends of transcripts in mRNA profiles (Figure 1C), which are a natural consequence of visualizing the 5′ ends of fragments produced via random fragmentation of multiple mRNA copies of any given transcript, we mapped 49 TSS upstream of 54 out of the 85 officially annotated genes (See Materials and Methods, Tables 1 and 2). The annotation of the TSS coordinates for the remaining 31 viral genes was impeded by low coverage or the presence of overlapping transcripts. Among the mapped viral genes, the discrepancy between the number of TSS and genes, stems from the existence of bi- and poly-cistronic mRNAs (Figure 3A). Of the 49 TSS mapped, 28 are novel while 21 correspond to annotated transcripts whose TSS were previously characterized. Of the 21 previously documented TSS described in our study, 13 are mapped exactly as in the literature and 8 are located within 50 nucleotides of their reported coordinates as previously resolved by 5′ rapid amplification of cDNA ends (5′RACE) (Tables 1 and 2). Interestingly, sequence alignment of the promoter regions corresponding to the TSS unveiled in our analyses, shows the presence of a TATA-box 30 nucleotides upstream of 41 TSS, which remarkably corresponds to the same location of this cis-regulatory element in human promoters. The remaining eight TSS are TATA-less (Table S1). Our observations clearly reflect the strict evolutionary dependence of the pathogen on the host's transcriptional machinery (Figure 3B) [29]–[31].

Like cellular messages, KSHV transcripts are protected by a 5′ 7-methylguanosine cap and a 3′ poly-adenylate (polyA) tail [32], [33]. To map functional polyadenylation signals (PAS) in the viral genome, we selected the RNA-Seq reads that contained a stretch of 5 or more adenosines at their 3′ end and, after trimming this poly-A sequence, aligned the reads to the KSHV genome. The 3′ positions of aligned reads were then marked as polyadenylation cleavage sites, except when the genome contained a complementary poly-T stretch at the same location as the poly-A stretch. Using this approach, we mapped 94 putative cleavage sites, corresponding to 42 transcripts and 74 genes (Tables 1, 2 and 3, Table S2). Our data, and recent studies mapping the 3′UTRs of KSHV genes [34], [35], support the existence of bi- and poly-cistronic messages, as well as transcript clusters with distinct transcription start sites (TSS) that end in a common PAS, suggesting the existence of alternative nested promoters upstream of such PAS's (Figure 3A, Table S2).

Interestingly, sequence analysis of a 60-nucleotide window centered on the predicted cleavage site for the polyadenylation machinery confirmed the presence of the canonical AAUAAA motif in 83% of mRNAs (35 out of 42), the alternative AUUAAA motif in 17% (7 out of 42), and the accompanying downstream GU rich element in all of these transcripts (Figure 3C and Table S2). Similar observations were recently reported in genome-wide analyses of polyadenylation sites in PEL cells infected with KSHV [36]. The presence of these conserved elements highlights once more the strict dependence of the virus on host factors that control the RNA processing of Pol II transcripts [37].

### Identification of viral, ribosome-protected non-coding RNAs by mRNA-Seq and Ribo-Seq

Besides the densely packed coding regions and regulatory features we annotated, our mRNA-Seq data also show the massive accumulation of sequence reads that map outside of previously annotated coding regions, thus indicating highly permissive transcription of most of the viral genome late in the lytic cycle (Figure S3). Two of these regions correspond to two long transcripts recently discovered by our group, the 10 kb antisense RNA to the latent transcripts (ALT) and the 17 kb K1-ORF11 antisense (K1/11-AS) [6]. Intriguingly, these long RNAs show short regions modestly populated by ribosomes, suggesting they may have a coding potential (Figure S4A and S4B).

**Figure 4:**
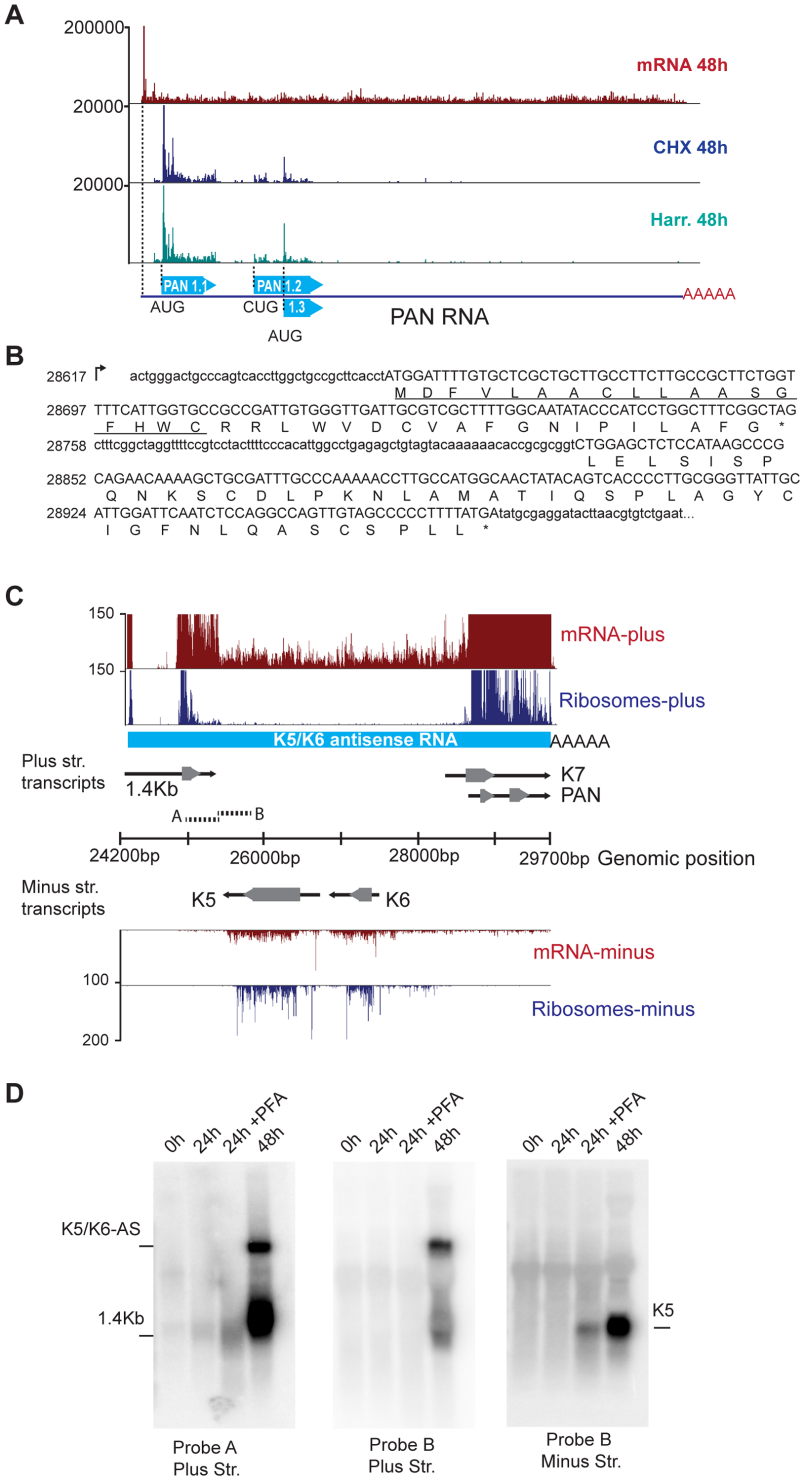
mRNA-Seq reveals ribosome-protected non-coding RNAs in KSHV. (A) The PAN transcript is protected by ribosomes during the lytic cycle. mRNA-Seq and Ribo-Seq of the PAN gene at 48 hr post reactivation. Ribo-Seq in cells treated with CHX (blue) or Harr (teal) show the accumulation of ribosomes on three distinct coding regions of this transcript. (B) PAN codes for three putative sORFs, PAN1.1 (37aa), PAN1.2 (44aa), and PAN1.3 (25aa), which can be identified as ribosome-populated regions (in capital letters). The predicted signal peptide in PAN1.1 is underlined. (C) The K5/K6 antisense transcript is devoid of nucleosomes following lytic reactivation. mRNA-Seq and Ribo-Seq of the genomic region from 24200 bp to 29700 bp at 48 hr post induction. The light blue solid line corresponds to the K5/K6 antisense RNA. Solid black arrows indicate transcripts and grey arrowheads indicate coding regions. (D) Northern blot for the same region depicted in (C). The A and B probes are indicated as short broken lines in (C). PFA: phosphonoformate. See also Figure S5 and Table S3.

It is noteworthy that this observation was not restricted to ALT and K1/11-AS. Surprisingly, our Ribo-Seq data also revealed the presence of ribosomes on the “non-coding” RNA PAN (polyadenylated nuclear RNA). PAN is the most abundant viral transcript during the lytic cycle and is required for viral gene expression and virion production [33], [38], [39]. Interestingly, and in spite of PAN's reported nuclear localization, we observed initiating ribosomes accumulating at the start codon in the harringtonine treated samples, elongating ribosomes throughout the body of the transcript in the CHX treated samples, and an accumulation of releasing ribosomes at the stop codon in the samples not treated with any translation inhibitor, starting at 8 hr following reactivation and throughout the lytic cycle (Figure 4A, S5A and S5B). Taking in consideration this pattern of ribosome protection, classical of coding regions [14], we identified three predominant sORFs hosted within the PAN transcript: PAN1.1 (37 aa, 28655), PAN1.2 (44 aa, 28831) and PAN1.3 (25 aa, 28888) (Figure 4B). Besides these, we also identified 3 minor sORFs at the 3′ end of PAN, with very low ribosome occupancy (data not shown). To evaluate the coding capacity of PAN, we calculated the ribosome release score (RSS) for the three main putative ORFs, PAN1.1, 1.2 and 1.3. The RRS is a metric that takes in consideration that ribosome protection within a coding region ends after the stop codon and that no ribosomes should be present at the 3′UTR of the transcript following an ORF. A recent report by Guttman *et al.* indicates that the RSS provides an indirect measure of translation that allows the reliable differentiation between coding and non-coding transcripts [40]. The RSS calculated for PAN1.1, 1.2 and 1.3 are comparable to those of known coding RNAs and are similar to scores previously determined for small ORFs within mammalian transcripts [40], further supporting the translation potential of PAN (Figure S5D, E). Notwithstanding the low translation efficiency of the three major PAN sORFs (0.05 at 8 hpi to 0.2 at 72 hpi), our mRNA-Seq and Ribo-Seq data suggest that, owing to the significant accumulation of the PAN transcript during the lytic cycle, the putative peptides encoded in these sORFs could be quite abundant. In fact, PAN RNA represented up to 92% of the total viral mRNA-Seq reads and the ribosome-protected RNA corresponding to the small PAN peptides represented up to 1.7% of the total cycloheximide Ribo-Seq reads (Table S3). Thus, our data strongly indicate that this transcript is available for ribosome binding, and that in addition to its documented functions as a “non-coding” RNA, PAN may also be a presumptive coding RNA. It is important to note that the putative coding regions for PAN are overlapping with the ORFK7 transcript. However, close inspection of the ribosome accumulation at the start codon of ORFK7 indicates that in order for the PAN peptides to be encoded by the K7 transcript, the translation efficiency of the internal peptides would need to be 1000 to 10000 times more efficient than that of the main ORF K7 (Figure S5C). Based on this observation and the vast number of reads seen for the mRNA and ribosome protected fragments in PAN1.1, 1.2 and 1.3, we conclude that these putative coding regions are within the PAN transcript.

We hypothesize that despite their minuscule size, the putative peptides encoded by PAN may be functional. Indeed, the number and characterized functions of such small peptides are continuously increasing, and there is overwhelming evidence in other viral systems, as well as in eukaryotic cells, for the abundance and relevance of small peptides [14], [21], [41]. Encouraged by these findings, we started to look for a possible function of the predicted peptides encoded by PAN1.1, 1.2 and 1.3. To such end, we used bioinformatics tools that included diverse motif finding and peptide-function prediction engines [42], [43]. Surprisingly, multiple independent analyses predicted a putative signal peptide in PAN1.1 (Figure 4B), thus suggesting that this peptide may traverse the secretory apparatus. Importantly, the sequence of PAN1.1 and the other small peptides predicted within PAN show 100% conservation at the nucleotide level between different isolates of KSHV (data not shown). Further studies of these putative gene products are underway.

Three additional regions show extensive mRNA-Seq coverage, particularly at 72 hr post reactivation. These are the antisense transcripts corresponding to ORFK5/K6, as well as the antisense transcripts for ORFK9-ORF62 and ORFK2 (vIL6)-ORFK4.2 (Figure 4C, data not shown). We confirmed the existence of a 6 kb RNA antisense to ORFK5/K6 by northern blotting (Figure 4D). This antisense RNA, which we have denominated K5/K6-AS, corresponds to the T6.1 RNA previously described by Taylor et al. [28]. Strikingly, K5/K6-AS is devoid of any initiating or elongating ribosomes, and therefore may represent a *bona-fide* long non-coding RNA in KSHV that is inherently distinct from PAN, ALT and K1-11AS (Figure 4).

### mRNA-Seq and Ribo-Seq unveil strategies used by the virus to expand its peptide repertoire

Like other herpesviruses, KSHV makes widespread use of the cellular mRNA splicing machinery [14], [44]. To confirm known splice junctions and discover new ones, we annotated the possible viral splice junctions in a genome-wide fashion by employing HMMSplicer and TopHat on the mRNA sequences that did not align to either the viral or the human genome [22], [23]. Our results confirmed the presence of 27 splice junctions, corresponding to one or more introns in 17 viral genes (20% of genes). These included the well-characterized splice variants observed in ORF50 and ORF57, as well as the multiple splice variants of K8 and K8.1 (Figure 1 and Table S4). The coordinates of the splice junctions annotated using our experimental data confirm those from previous reports (Table S4), affirming the reliability of our combined methods.

**Figure 5:**
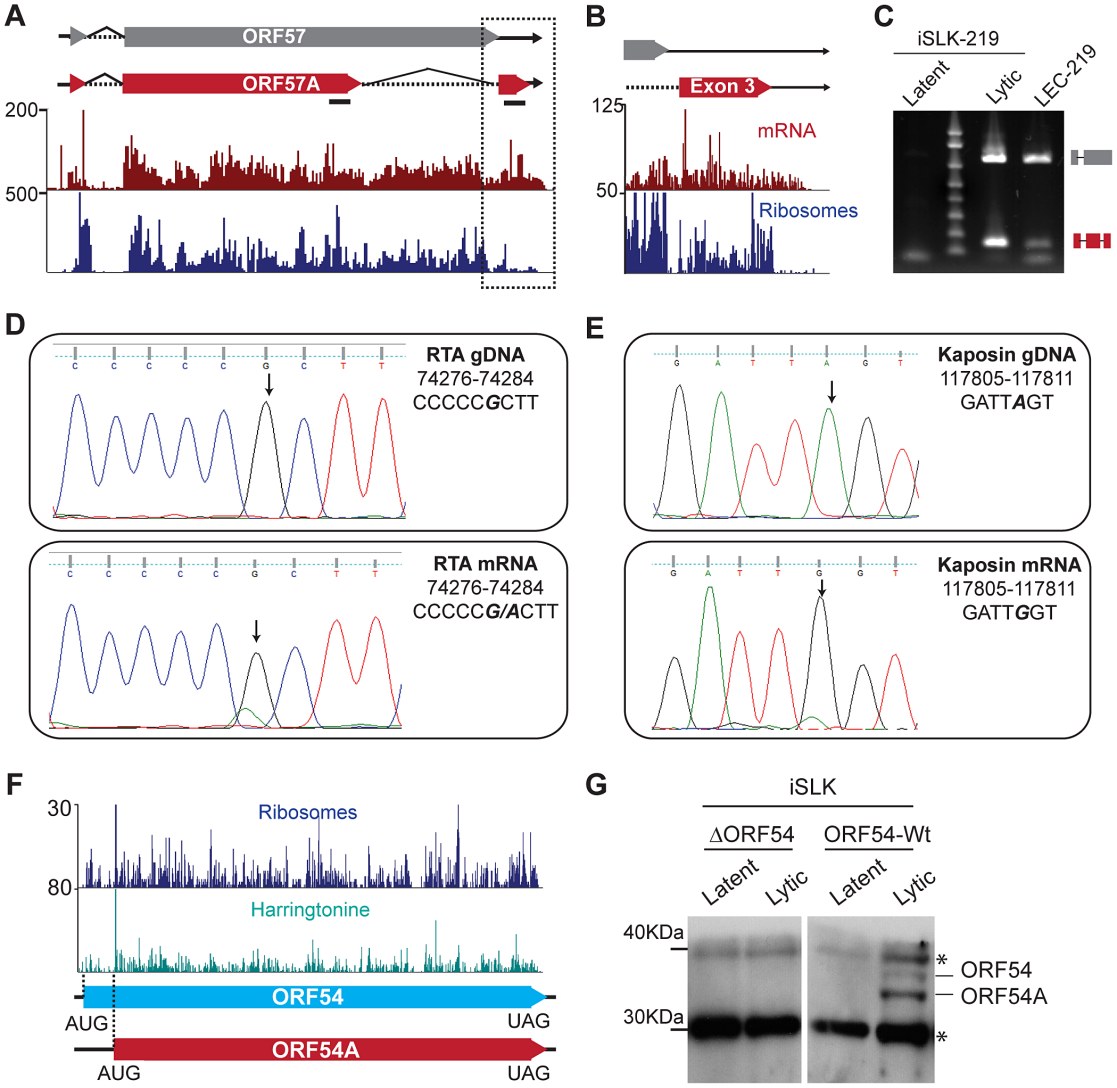
KSHV employs multiple strategies to expand and regulate its coding repertoire. (A–C) Novel splice variant of ORF57 detected by mRNA-Seq. Tracks for mRNA-Seq and Ribo-Seq in CHX treated cells (48 hpi) show the new, shorter-splice variant of ORF57, ORF57A (red box). Splicing of the second intron in ORF57 removes the UAA stop codon, resulting in the generation of a 33 aa C-terminal exon. (B) Zoom in of the region highlighted in (A). Note that ribosomes accumulate downstream of the ORF57 stop codon, indicating that the new exon may be translated. (C) Verification of ORF57A splicing by end-point PCR in lytic iSLK-219 (72 hpi) and LEC-219 cells. The primers used for amplification flank the intron boundaries and are shown in panel (A) as solid black lines. The high molecular weight product (∼800 bp) corresponds to the unspliced form of ORF57. The low molecular weight product (165 bp), corresponds to ORF57A, from which the second intron has been removed. (D–E) mRNA editing in the RTA and Kaposin transcripts. Sanger sequencing of RTA (D) and Kaposin (E) genomic DNA (top panel) and cDNAs (48 hpi) (bottom panel) from iSLK-219 cells. The edited nucleotide is bold, italic font. (F–G) ORF54 translation starts from two alternative initiation codons. (F) Ribo-Seq tracks for cycloheximide (CHX blue) and harringtonine (Harr teal) treated cells at 72 hpi. The dotted lines indicate the two translation initiation sites used for ORF54 translation. (G) Immunoblot for ORF54 in latent and lytic (48 hpi) iSLK cells, infected with Wt KSHV or an ORF54 knock-out virus (ΔORF54). *Non-specific product. See also Figures S6, S7 and S8.

Notably, our data not only correctly annotated known splice junctions but revealed 7 novel ones, thus increasing the number of splicing events from 20 to 27 (Table S4). One such splice junction is located at the 3′ end of the ORF57 transcript. ORF57 is a well-characterized KSHV protein thought to be an activator of mRNA maturation and transport, enhancing viral gene expression [45], [46]. Our data support the existence of canonical splice donor and acceptor sites in the new predicted junction (Figure S6A), which give rise to a novel splice variant of ORF57 in which the truncation of a second exon results in the accumulation of ribosomes on a previously uncharacterized third exon (Figure 5A,B). We confirmed the second splicing event of the ORF57 transcript by end-point PCR in iSLK (iSLK-219) and lymphatic endothelial cells (LEC-219) infected with recombinant KSHV.219, but not in infected B cells (BCBL-1) (Figure 5C and S6B).

The second splicing event in the ORF57 transcript results in the removal of a 571 nucleotide fragment encoding amino acids 266–455 within the second exon and leads to the loss of the canonical UAA stop codon of ORF57, resulting in the generation of a novel isoform, here named ORF57A, with a different C-terminus that contains 33 amino acids (Figure 5B). Interestingly, the stop codon of ORF57A (position 83464) is located downstream of the canonical polyA cleavage site in ORF57 (position 83453), suggesting the presence of a transcript with an extended 3′ end. We confirmed the existence of such longer mRNAs in iSLK-219 and BCBL-1 cells by PCR using a primer set annealing within the ORF57 and ORF57A coding regions, and downstream of the annotated polyA cleavage site (Figure S6C). ORF57A, the new alternative splice variant of ORF57, is 299 amino acids in length and lacks the C-terminal leucine zipper (aa 343–364), the second arginine-glycine-glycine rich domain (RGG) (aa 372–374), the zinc finger domain (aa 423–432) and the glycine-leucine-phenylalanine-phenylalanine (GLFF) domain (aa 447–450) (Figure 5A). While the expression of a truncated form of ORF57 could have functional implications, it is important to note that the ORF57A splice variant was detected only in cells infected with the recombinant KSHV.219 virus (iSLK-219 and LEC-219), and could reflect a secondary effect of the insertion of the GFP/RFP reporter cassette downstream of ORF57 [18], leading to the activation of this cryptic splice site within the ORF57 transcript. This observation demonstrates that elements inserted within the viral genome, even in regions that are seemingly devoid of regulatory/functional elements, may not be inert and could have repercussions on viral gene expression and/or function.

A second posttranscriptional mechanism employed by KSHV to expand its coding capacity is mRNA editing. The post-transcriptional recoding of RNA results in single nucleotide discrepancies between the genomic and transcript sequences [47], [48]. By comparing our mRNA-Seq and DNA-Seq data sets, we found 6 instances of mRNA editing in KSHV in two or more time points (Table S5). Three of such editing events include the previously reported A-to-G transition in genomic position 117,809 within the transcript encoding Kaposin [49] and two novel G-to-A transitions at genomic positions 72,795 and 74,281 of the mRNA encoding RTA, leading to amino acid substitutions in the corresponding encoded polypeptides (Table S5). We confirmed these editing events by end-point PCR amplification of cDNA followed by Sanger sequencing (Figure 5D, E, data not shown). Interestingly, we note that the Kaposin message is edited starting at 24 hr following reactivation, and that the relative amount of edited transcript increases dramatically as the lytic cycle progresses, leading to a highly penetrant A-to-G transition at 72 hr (Figure 5E, Figure S7A and B). The surge in the levels of Kaposin mRNA editing is concomitant with the up-regulation of all isoforms of the adenosine deaminase acting on RNA (ADAR), the enzyme implicated in A-to-I editing (Figure S7C). Our observations indicate that ADAR is at least partially insensitive to the generalized host shutoff mediated by the viral endonuclease SOX (Figure S7D) [50]. The consequence(s) of higher levels of ADAR on host mRNA and other viral transcripts, if any, remain to be determined. In addition to investigating the mechanisms of regulation and the activity of ADAR during lytic infection, it would be of great interest to ascertain the biological impact of the A638T substitution in RTA. The post-translational modification prediction tool NetPhosK [51] suggests that such a mutation improves the sequence context for S634 and S636 phosphorylation in the C-terminus of the protein [52]. The biochemical and functional consequences of this mRNA editing in RTA are yet to be determined and will be the focus of future studies. Two of the predicted events, a U-to-G transversion in position 6144 within ORF6 and a G-to-U transversion in position 96434 within ORF59, were not affirmed by Sanger sequencing, highlighting the importance of validation of putative mRNA editing sites identified through next-generation sequencing (data not shown).

A third mechanism used by KSHV to increase the coding capacity of its viral genome is independent of transcriptional control and involves the manipulation of translation. Ribo-Seq allowed us to accurately map most of the annotated viral ORFs while affording the opportunity to discover several dozen undocumented peptides and putative protein isoforms (Figure 2B). Our data show five coding regions that are of particular interest in that regard. ORF70, ORF K6, ORF54, ORF62 and Kaposin exhibit a remarkable accumulation of initiating ribosomes on multiple in-frame translation start sites, strongly arguing in favor of the presence of at least two protein variants for each one of these ORFs (Figure 5F, Tables 1 and 2, Table S6, Figure S8, File S1 and data not shown). We examined the expression of one of these proteins, ORF54 by immunoblotting in lysates from latent and lytic iSLK cells infected with Wt KSHV or an ORF54 deleted virus [53]. In perfect agreement with our Ribo-Seq data, we detected two isoforms of ORF54 using an antibody directed against the C-terminus of the protein. These migrate at ∼ kDa (318 aa -ORF54) and 32 kDa (291 aa-ORF54A) in denaturing SDS-PAGE gels, consistent with our finding of 2 polypeptides that share a common C-terminal domain but possess distinct amino-termini owing to the usage of alternative translation initiation sites. Furthermore, our sequencing data indicate that the previously uncharacterized short form of ORF54 is the most abundant one (Figure 5F), which is also in exact agreement with our immunoblot analysis (Figure 5G). The ORF54 and ORF54A products detected during lytic infection of iSLK cells are also clearly seen in HEK293 cells transfected with C-terminally tagged versions of the gene (data not shown). Taken together, our data affirm that KSHV can selectively use alternative start codons to amplify the peptide repertoire synthesized during the lytic cycle.

### Ribo-Seq reveals a cryptic translational regulatory network

The peptide coding capacity of KSHV has been defined globally employing *in silico* approaches and proteomics studies [4], [11] and at a single-gene level by mutagenesis, epitope-tagging and immunodetection. We sought to obtain a unifying and comprehensive understanding of the viral peptide coding capacity using ribosome profiling. Using Ribo-Seq, we mapped most of the previously annotated viral ORFs with precision and, remarkably, discovered 63 new ORFs, representing a higher than 45% increase of the annotated coding capacity of KSHV to date. The vast majority of these new ORFs encode peptides smaller than 100 amino acids and, in 44% of the cases, peptides that are translated from initiation codons with consensus or near-consensus Kozak sequences [54]. Thus, we have reclassified the coding regions of KSHV into primary ORFs, alternative splice variants, internal ORFs, ORFs with alternative start codons, small (sORFs) and upstream ORFS (uORFs) (Figure 2, Tables 1 and 2, Figure S9B–C, File S1).

We defined sORFs as all of those regions encoding peptides of ∼100 aa or less that are not found at the 5′ of an annotated viral gene [55]. In total we found 14 sORFs within 6 transcripts (Table S7). Among these, we clearly detected ribosomes populating the 5′ end of the ORF50-antisense (50-AS) transcript at 24–48 hr post reactivation (Figure S9B), confirming recent reports that indicate that this mRNA is indeed present in polysomal fractions [7]. While previous transfection-based studies from our lab and others have characterized peptides ranging from 17 to 48 amino acids starting from multiple AUG initiation codons, our Ribo-Seq data indicate that the accumulation of initiating ribosomes in an authentic viral infection involves at least three non-canonical start codons giving rise to small peptides from 8 to 76 aa [7], [8].

The second class of small coding regions revealed by our Ribo-Seq data consists of a group of 36 upstream ORFs (uORFs). These uORFs are present in the leader sequence of annotated ORFs and encode peptides of ∼100 aa or less [56]. We noted that uORFs are very numerous and widely distributed across the whole genome (Table S7). In total, 24 genes have between 1 and 6 uORFs that are either in-frame or out-of-frame with the main ORF (Figure S9C). Interestingly, and as has been previously reported for HCMV and mammalian cells [14], [21], 44% of uORFs are translated from a non-canonical start codon and are highly detected at late times during reactivation (Figure S9A, Table S7).

**Figure 6:**
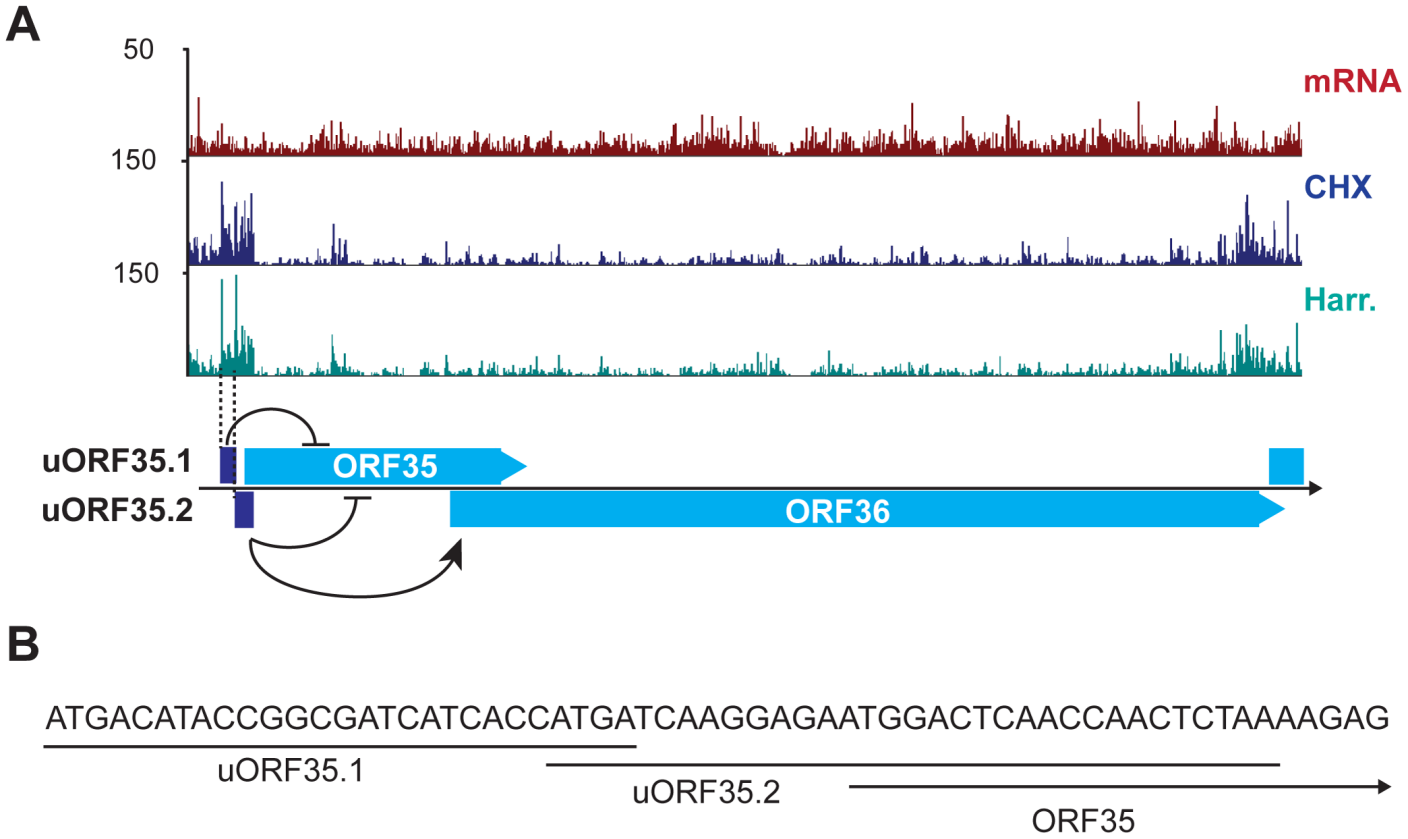
KSHV encodes functional uORFs. (A) mRNA-Seq and Ribo-Seq tracks (CHX and Harr) show the accumulation of ribosomes in the regions of uORF35.1 and uORF35.2. These uORFs regulate the expression of ORF35 and ORF36 (adapted from [57]). (B) Start and coding sequences for uORF35.1, uORF35.2 and ORF35. See also Table S7.

An example of the regulatory capacity of uORFs in KSHV was recently documented by Kronstad and colleagues, who described the functions of two uORFs identified in our Ribo-Seq data as uORF35.1 and uORF35.2 (Figure 6A) [57]. uORF35.1 and uORF35.2 have opposing regulatory functions on the translation of the downstream ORFs ORF35 and ORF36, in a mechanism akin to that described for eukaryotic uORFs regulating cell-stress response genes [58]. These uORFs are located in the 5′ leader sequence of the ORF35–36 bicistronic transcript. The uORF35.1 small peptide (8 aa) is in-frame with respect to ORF35, while the uORF35.2 small peptide (10 aa) is an out-of-frame overlapping ORF with respect to ORF35 (Figure 6B). Both of these uORFs inhibit the expression of ORF35, as their deletion promotes accumulation of this protein. However, uORF35.2 has stimulatory effects on the translation of the most 3′ gene, ORF36, via a continuous scanning mechanism [57]. These data affirm the existence and functional significance of two of the uORFs identified by Ribo-Seq, and support the reliability of this method for identifying such elements.

### The time-dependent phase switch controlling gene expression in KSHV

**Figure 7:**
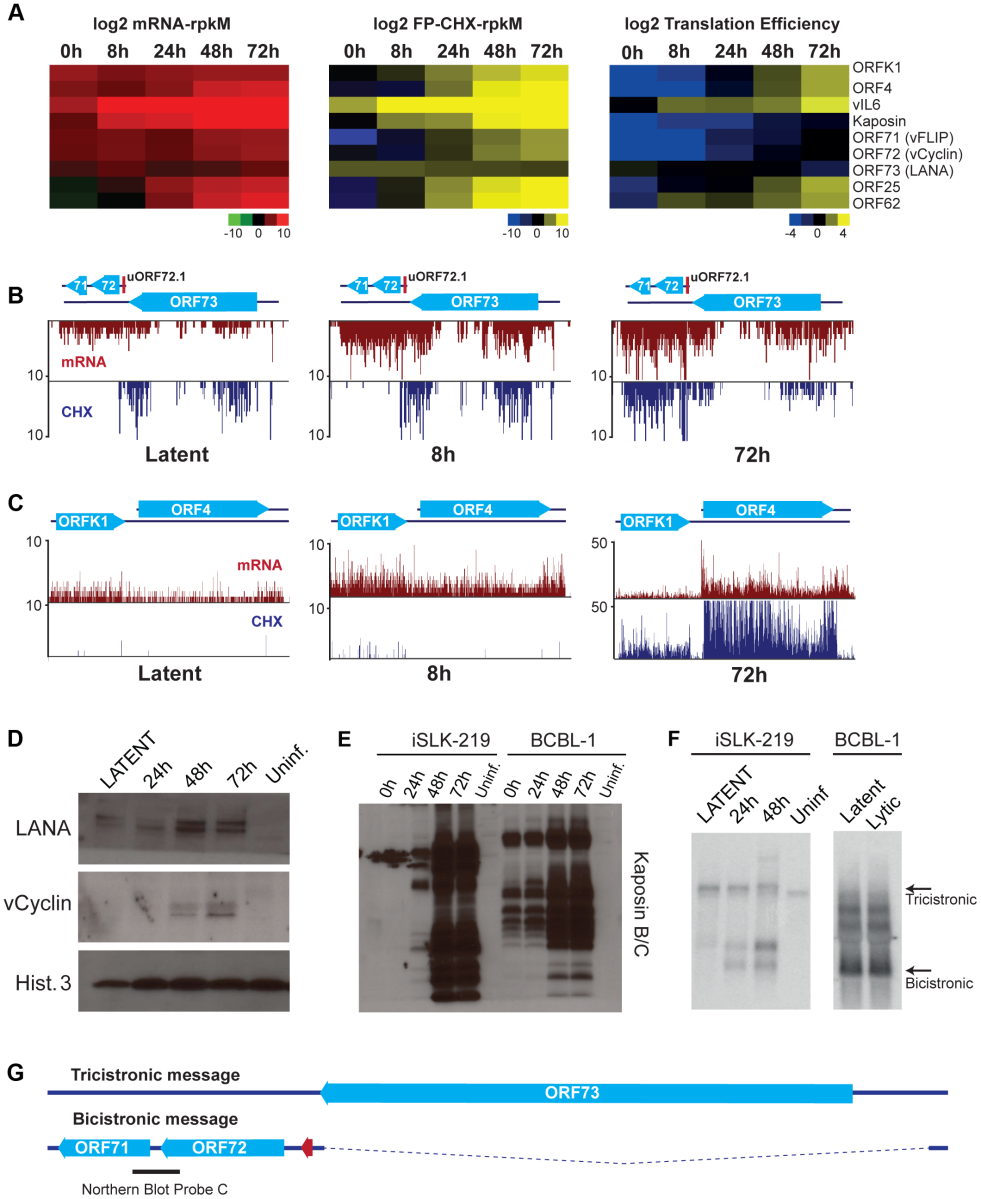
Gene expression is limited during latency in iSLK-219 cells. (A) Log_2_ of normalized mRNA-Seq, Ribo-Seq counts (reads per kilobase million, rpkM) and translation efficiency (TE = Ribo-seq rpkM/mRNA-seq rpkM) for the latent transcripts and two late lytic ORFs (ORF25 and ORF62). Note the low TE for the latent genes at 0 h and 8 h. (B–C) Ribosome occupancy of latent transcripts changes with time. mRNA-seq (red) and Ribo-seq (CHX blue) for (B) the latency locus and (C) the K1-ORF4 region. (D–E) Immunoblot for the products of the latent transcripts (D) LANA and vCyclin, and (E) Kaposin. (F) Northern blot for the tricistronic (LANA-vCyclin-vFLIP) and the bicistronic (vCyclin-vFLIP) illustrated in (G). mRNA (100 ng-iSLK-219) or total RNA (10 ug-BCBL-1) were probed for vCyclin and vFLIP (see black bar in G). (G) Schematic of the tricistronic and bicistronic transcripts in the major latency locus.

**Figure 8:**
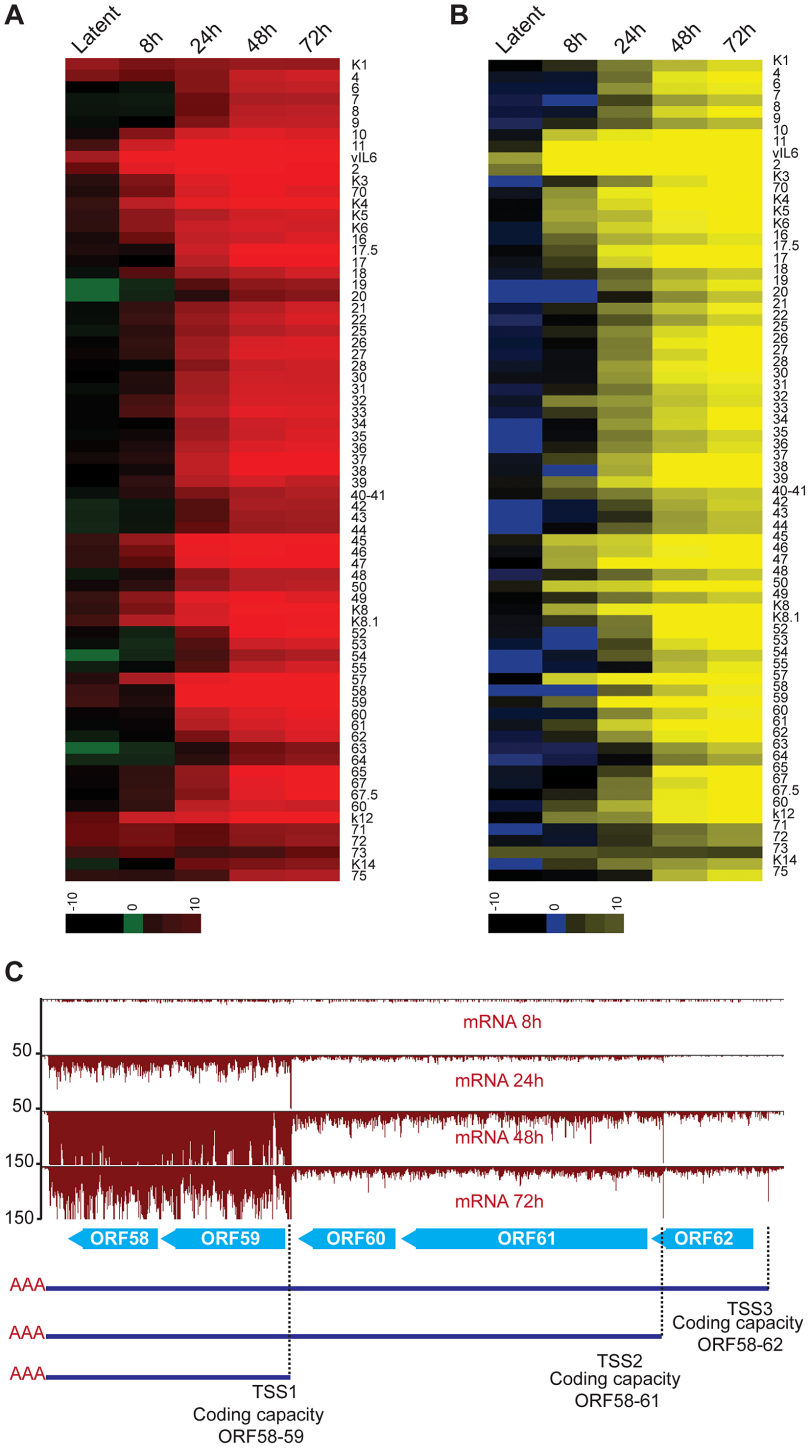
Lytic reactivation is subject to a transcriptional timer. (A–B) Log_2_ of normalized mRNA-Seq (A) and Ribo-Seq (B) counts (reads per kilobase million, rpkM) was calculated for the primary ORFs. The log2 scores reflect mRNA-expression (mRNA-seq) or ribosome occupancy (Ribo-seq) for each gene. The ORFs are organized by genomic position from ORFK1 to ORF75. The ORFs that were not annotated in our study have not been included in the plots (C) mRNA-Seq for the ORF58–62 locus. Three TSS sharing a single PAS were mapped in this region. Note the timing of expression for each of the transcripts.

The phase switch from latency to the lytic cycle is a highly regulated process that requires the temporally controlled expression of genes. Our strategy of mapping transcripts and coding sequences across different stages of the lytic cycle in an RTA-regulatable expression system revealed a built-in timer for viral reactivation that relies on the use of specific TSS during the distinct stages of the viral life cycle. Fine temporal mapping of viral transcription include 4 latent messages, 13 early genes that are expressed starting at 8 hr, 19 genes expressed between 24 hr and 48 hr after reactivation and 38 genes at 48–72 hr following DNA replication (Tables 1, 2 and 3, Figure 7A, Figure 8A and 8B). We took advantage of the restricted latency and protracted lytic cycle observed in iSLK-219 cells when compared to cells of lymphoid origin (unpublished observations) to study the kinetics of viral transcription in much finer detail. As expected, our results show that only a handful of transcripts are expressed during latency, namely the K1-ORF4 bicistronic message, vIL6, Kaposin and the LANA-vCyclin-vFLIP tricistronic transcript. Interestingly, the ribosome profiling of latent cells shows that only vIL6 and LANA are protected by ribosomes (Figure 7A and 7B). Furthermore, we confirmed by immunoblot the presence of LANA and the absence of Kaposin and vCyclin proteins in latently infected cells (Figure 7D and 7E). These observations raised questions about whether the K1, vCyclin and vFLIP proteins might be importantly regulated at the level of translation. Consistent with this, the Kaposin, K1, vCyclin and vFLIP transcripts are abundantly protected by ribosomes upon induction of the lytic cycle, and their cognate proteins can be detected by immunoblotting after such induction (Figure 7C, 7D and 7E). However, subsequent Northern blot analysis revealed the pattern of accumulation of vCyclin and vFLIP transcripts in iSLK differs from that previously observed in B cells (Figure 7F and 7G) [59]. To our surprise, we could detect only the tricistronic (LANA-vCyclin-vFLIP), but not the bicistronic (vCyclin-vFLIP) message, in latent iSLK-219 cells (Figure 7F). However, the bicistronic transcript, which has previously been proposed to be the mRNA for these 2 proteins, is abundantly expressed in lytic iSLK-219 cells (Figure 7F) [60]. This observation, in combination with our mRNA-seq and Ribo-seq data, suggests that vCyclin and vFLIP proteins are indeed mainly expressed from the bicistronic message and that their expression is primarily regulated at the RNA level during latency in iSLK-219 cells. It remains possible that translational control governs the latent expression of ORF K1.

As to the lytic cycle, our data support that most gene expression during this phase is controlled through transcriptional regulation, as the vast majority of the newly synthesized mRNAs are protected by ribosomes without delay (Figure 8A and 8B). A notable example of the temporal selection of distinct TSS can be seen in the transcription of the ORF58–62 locus which encodes the EBV-BMRF2 homologue (ORF58), the DNA polymerase processivity factor (ORF59), the small (ORF60) and large (ORF61) subunits of the viral ribonucleotide reductase, and a small capsid protein (ORF62) (Figure 8C) [61], [62]. In this case, our data clearly distinguish at least three independent transcripts with different expression kinetics: the RNA of ORF58–59 is expressed first, followed by the delayed early ORF60–61, and the late ORF62 transcript. The differential expression of these mRNAs correlates with their biological function, as genes required for DNA replication (ORF59, 60 and 61) are expressed before structural proteins (ORF62). Through the detection of these three distinct cistrons, our results unequivocally support the existence of three independent promoters that are integral part of the aforementioned temporally-regulated gene activation. Indeed, our TATA-box analyses, as well as previous reports, have identified at least three temporally-regulated promoters in this region (Table S1), supporting the differential expression control of ORF58–62. The time regulated selection of TSS has also been reported in HCMV, where the expression of transcripts from alternative TSS results in the translation of different protein products at specific times during infection, and represents a conserved mechanism of gene regulation in herpesviruses [14]. Taken together, our results, alongside previous studies, suggest that the transcription of KSHV genes is tightly regulated by promoter availability and the dynamic interplay of host and viral transcription factors in a time-dependent phase switch operating in the transition from latency into the lytic cycle [63], [64].

## Discussion

The wealth of information we generated by combining our DNA-Seq, mRNA-Seq and Ribo-Seq datasets allowed us to build a comprehensive, high-resolution map of the viral genome over the KSHV life cycle. Our approach showcases the great analytical power of next-generation sequencing technologies, as we were able to pan-genomically map coding, non-coding, and regulatory features of the KSHV episome in iSLK-219 cells. Most importantly, this study provides tangible evidence derived from experimental data, as opposed to *in silico* prediction approaches, of the mechanisms employed by herpesviruses to widen the coding capacity of their genome through the use of diverse strategies including splicing, mRNA recoding, and alternative start codon usage. Furthermore, we demonstrate that the viral genome is not a conventional source of coding sequences as traditionally defined by *in silico* predictions and homology analyses, but a rather rich collection of diverse coding sequences that include numerous viral sORFs and uORFs. Some of those may exert translational modulation of other viral ORFs, thereby enriching and adding complexity to the viral gene regulatory profile. It is noteworthy that several dozen viral features uncovered by our group in this study were not documented earlier because of the limitations imposed by conventional methods employed to study gene expression.

Our results confirm the striking conservation of eukaryotic cis-regulatory elements in the KSHV genome, including TATA boxes, polyadenylation signals, and splice junctions. The conservation of both, the sequence and position of these features reflects the strict dependence of the virus on host factors and importantly, imposes a major constraint for the pathogen because of the need to compete with host factors for transcription and translation of virally-encoded products. To circumvent this competition, the virus causes the gradual but massive inhibition of host protein expression by increasing mRNA turnover. This mechanism, known as host shutoff, is orchestrated by the concerted action of the viral endonuclease SOX and the cellular exonuclease Xrn1 [65]. The elimination of competing cellular mRNAs renders the translational machinery components available to the virus, relieving the restrictions with respect to the expression of viral gene products. In addition to this upsurge in the availability of translation factors caused by the decreasing amounts of host transcripts, KSHV also hijacks essential cellular pathways that directly impinge on translation. Notable examples are the virus-orchestrated activation of mTOR and MAP kinases, which promote translation during the lytic cycle and result in the expression of viral ORFs required for the progression of infection [66]–[68]. It thus follows that the tug of war between host and virus would result in the commandeering of essential cellular factors by the virus to promote its own replication. Based on our analyses, we posit that the increased accessibility to ribosomes, the creation of an environment that is conducive to high translation, and the accumulation of viral transcripts results in an extremely favorable scenario for pervasive translation of viral encoded ORFs herein annotated, including sORFs and uORFs, and the short ORFs in PAN. Future studies in cells infected with viruses defective for host shutoff will help clarify the contribution of this mechanism to the viral translational output.

Another striking yet puzzling finding derived from our data is the protection of PAN by initiating and elongating ribosomes. This well-characterized and abundant viral RNA is expressed during the lytic cycle and has a predominant nuclear localization [33], [38]. Surprisingly, this nuclear RNA is clearly bound by ribosomes, indicating that either a fraction of PAN is cytoplasmic and available for translation, or (less likely) that ribosomes can access PAN in the nuclear compartment [69], [70]. Even more remarkable is the fact that the relative abundance of ribosome-bound PAN fragments suggests that the encoded peptides, if stable, could be abundant. Irrespective of their abundance, the biological functions of such putative peptides remain to be investigated. Bioinformatics analyses failed to identify discernible domains or particular motifs in 2 out of the 3 PAN-encoded peptides identified in our study. We did however find recognizable motifs within the primary structure of PAN1.1. This peptide harbors a putative signal sequence but lacks a discernible transmembrane domain or an ER retention signal, thus suggesting that PAN1.1 may be a secreted product. Studies aimed at the identification and characterization of PAN1.1 products are currently ongoing.

An interesting observation from our analyses is the detection of mRNA editing in two viral transcripts. This posttranscriptional mechanism of coding and non-coding RNA editing is conserved in eukaryotes. In humans extensive RNA editing has been reported, the majority of the events corresponding to A-to-I transitions mediated by the family of deaminases ADAR. Indeed, alterations in the activity of these enzymes are associated with disease [48], [71]. RNA editing is not restricted to eukaryotic messages and has been observed on viral transcripts in cells infected with RNA and DNA viruses [72]. The effects of RNA editing of viral transcripts antagonize (HCV, MV), or promote (HIV, HDV) viral activity and may affect the function of particular non-coding RNAs (EBV) or viral proteins (KSHV). As in humans, most of the RNA editing events detected on viral transcripts are ADAR dependent and correspond to A-to-I transitions, as the one we observe in Kaposin (position 117809). Interestingly, we see the protein levels of ADAR1 increasing throughout the viral lytic cycle, indicating the message for this enzyme partially escapes the widespread host shutoff caused by SOX. It is not clear however what would be the effects of such up-regulation of ADAR on RNA editing of host messages, given that a large number of transcripts are degraded during KSHV lytic infection. In addition to the nucleotide change in Kaposin, we also detect the non-canonical G-to-A editing in RTA (positions 72795 and 74801). While rare and less frequent than the A-to-I change, the G-to-A recoding has been previously identified in HIV and prostate and colorectal cancer [73], [74]. The enzyme responsible for this editing event has not been characterized to date, and KSHV lytic infection may provide a useful system for its identification.

Remarkably, our results strongly suggest that the peptide coding capacity of KSHV has been previously underestimated. This is illustrated in the overall high representation of sORFs and uORFs in the viral genome, often translated from near canonical start codons. The functional implications of such translation initiation events have been documented in several eukaryotes (yeast and metazoans) where translation initiation from near canonical codons under physiological conditions occurs more often than anticipated [21], [75]–[77]. In the case of KSHV, it is tempting to speculate that the increased use of non-canonical start codons, particularly late during infection (48 and 72 hr), is a probable consequence of the high availability of ribosomes and the translational permissiveness observed during the lytic cycle. A plausible explanation for the non-canonical start codon selection could be the abundance of eukaryotic translation initiation factors (eIFs) such as eIF1 and eIF5. It has been shown that these two proteins have opposite effects in the selection of the start codon; eIF1 increases the stringency of codon selection favoring initiation from AUG initiator codons whereas eIF5 favors translation from non-AUG codons and AUG codons nested within a poor sequence context [78], [79]. The relative abundance and stability of these factors has not been characterized in KSHV-infected cells. One could propose that the ratio of these factors may change during reactivation, favoring the translation from non-AUG codons. Future studies exploring the sequence context of the start codons of viral sORFs, uORFs, and alternative variants of main ORFs, as well as studies aimed at uncovering the interplay between eIF1 and eIF5 during infection will undoubtedly shed light on the mechanisms governing the intricate translation patterns we observed during the KSHV lytic cycle.

Another surprise revealed in our studies was the abundance of uORFs encoded in the 5′ ends of viral transcripts. Like sORFs, uORFs have been found in eukaryotes, where they serve as modulators of gene expression during cellular stresses [80]. Their role has been extensively characterized in the face of amino acid starvation in yeast, where the accumulation of the transcription factor GCN4 depends on regulatory uORFs [81], [82]. This regulatory mechanism is evolutionarily conserved. In metazoans, uORFs regulate the abundance of the stress-responsive transcription factors C/EBPa/b, ATF4 and CHOP as well as the regulatory subunit of protein phosphatase 1, encoded by the GADD34 gene [58], [83], [84]. Although the role of uORFs in viruses remains largely unexplored, these regulatory elements permeate many viral families [14], [85]–[87] suggesting they may also control viral gene expression in instances where cellular stress pathways are engaged. In KSHV, the translational regulatory function of uORFs controlling the expression of ORF35 and ORF36, has been recently described [57]. The existence of a plethora of uORFs throughout the viral genome strongly indicates that this mechanism may be more widely used by KSHV than previously suspected.

Taken together, our results illustrate the dynamics of gene regulation during the different stages of the KSHV life cycle, while they also reveal that the coding capacity of its genome goes well beyond what was anticipated by *in silico* analyses. The striking conservation of the mechanisms employed by host and virus to regulate transcription, translation, and the diversity of the peptide repertoire, elevates KSHV as a valuable model system to explore the mechanistic underpinnings of the host-virus interactions in herpesviruses at large, as well as those of fundamental cellular processes, including the control of translation initiation in response to cellular stress.

## Materials and Methods

### Cell lines

iSLK and iSLK-219 cells (kindly provided by JinJong Myoung) were maintained in DMEM supplemented with 10% fetal bovine serum, L-glutamine (2 mM, Invitrogen), penicillin (100 IU/ml, Gibco) and streptomycin (100 ug/ml, Gibco) at 37°C under a 5% CO_2_ atmosphere. iSLK-219 cells were grown in the presence of puromycin (10 mg/ml, Invivogen) to maintain selection for the viral episome. LEC-219 cells were maintained in EBM-2 (Lonza cc3156) media supplemented with the EGM2-MV kit (Lonza cc3203) in presence of 0.25 ug/ml puromycin to maintain selection for the viral episome. BCBL-1 cells were maintained in RPMI supplemented with 10% fetal bovine serum, L-glutamine (2 mM, Invitrogen), penicillin (100 IU/ml, Gibco) and streptomycin (100 ug/ml, Gibco) at 37°C under a 5% CO_2_ atmosphere.

### Induction and assessment of lytic replication in iSLK-219 cells

To induce entry to the lytic cycle, iSLK-219 cells were seeded at 1–2.5×10^5^ cells/ml and 24 h after seeding (∼70–80% confluent) cells were induced with doxycycline (1 µg/ml, BD Bioscience cat 631311,). To prevent viral DNA replication in the cells collected at 24 hpi, we induced these cells with Dox in the presence of phosphonoformate (500 uM). All other time points were treated with Dox alone. At the indicated times after induction viral reactivation was evaluated by microscopy detection of the PAN-RFP reporter. To determine the timing of KSHV DNA replication, DNA was isolated at the indicated times following reactivation using the DNeasy blood and tissue kit following manufacturer guidelines (Qiagen cat 69581). The DNA (20 ng) was used for qPCR using primers for the LANA promoter (Fwd: 5′ AGGATGGAGATCGCAGACAC 3′; Rev: 5′ CCAGCAAACCCACTTTAACC 3′) or GAPDH as a normalization control (Fwd: 5′ AGCCACATCGCTCAGACAC 3′; Rev: 5′ TGGAAGATGGTGATGGGATT 3′).

### Immunoblotting and antibodies

Cells were washed and collected in RIPA buffer (10 mM Tris pH 8; 1 mM EDTA; 150 mM NaCl; 5% glycerol; 0.1% sodium deoxycholate; 0.1%SDS; 1%Triton x-100) at the indicated time points. Cell lysates were clarified and protein concentration was quantified using the Bio-Rad DC protein assay following manufacturer guidelines. 10 ug of protein per sample were fractionated by Bis-Tris PAGE (4–20% gels in MES SDS-running buffer) and transferred to nitrocellulose membranes. Immunoblots were processed, incubated with primary antibody overnight and developed using ECL reagent according to manufacturer recommendations.

The following antibodies were purchased from commercial suppliers: K8.1 (Advanced biotechnologies cat. 13-213-100) Actin (Sigma-Aldrich cat. A2228), ADAR1 (Abcam cat. ab126745), vCyclin (SCBT cat. 19415) and K-bZIP (SCBT cat. F33P1). The LANA rabbit polyclonal antibody was raised against a synthetic peptide from the acidic domain of LANA (Polson and Ganem, unpublished). The Kaposin B and C antibody was raised against the DR1/DR2 regions of the protein (Bisson and Ganem, unpublished). The ORF54 rabbit polyclonal antibody was raised against a synthetic peptide from the C-terminal region of the protein (aa 280–298 EDTNSVRKHTNEDNPVHEP) (Covance).

### Preparation of ribosome profiling samples

Ribosome profiling was performed as previously described [13], [21] with some modifications. In brief, iSLK and iSLK-219 cells were left untreated, or treated with cycloheximide (100 ug/ml, 1 minute) or harringtonine (1 ug/ml, 90 seconds) followed by cycloheximide (100 ug/ml, 1 minute). After treatment cells were washed in cold PBS twice and lysed in lysis buffer (20 mM Tris, 1% triton, 220 mM NaCl, 15 mM MgCl2, 1 mM DTT, 8% glycerol) supplemented with Turbo DNAse (Ambion cat. AM2238). The lysate was clarified, treated with RNAse I (Ambion cat. 2295) and overlaid on a 34% sucrose cushion. Monosomes were isolated by centrifugation at 69000 rpm for 4 hr in a TLA 110 rotor. Ribosome protected RNA fragments were isolated from the monosomal fraction by acidic-phenol extraction.

### Total RNA extraction and polyadenylated RNA selection for mRNA-Seq

Total RNA was extracted from 1×10^7^ cells using RNABee (AMSBIO cat. Cs-104B) following the manufacturer guidelines. Polyadenylated RNA was isolated from the total fraction using Oligotex mRNA kit (Qiagen cat. 70022). The resulting mRNA was partially fragmented by alkaline hydrolysis with sodium carbonate to ∼150 nt segments on average, and then fragments between 40–100 nt were isolated from gel. The laboratory of Jonathan Weissman has previously documented that this partial fragmentation results in the preferential accumulation of mRNA 5′ terminal fragments for most transcripts with a non-overlapping transcription start site [14]. We determined that for the transcripts where we were able to annotate a transcription start site, there is a 4–5 fold enrichment (average 5-fold, median 4-fold) of the number of reads for the first 10 nt of the transcript compared to three 10 nt windows within the gene body (nt 20–30, 30–40, 50–60 data not shown). The mRNA profiles in all figures show counts of the 5′-most bases of sequencing reads.

### Library generation, sequencing, sequence alignments, normalization, and splice junction discovery

Strand specific libraries were generated as in Ingolia et al., 2012, with the modifications described in Stern-Ginossar et al., 2012. Samples were sequenced on either the Illumina Genome Analyzer II or the HiSeq 2000 using the truseq sbs kit v3 50 cycles (Illumina cat. FC-401-3002). Sequence analysis was done as described in Stern-Ginossar et al., 2012. Briefly, linker and polyA sequences were removed from the 3′ end of the reads preceding the alignment. Sequencing reads were aligned with Bowtie2 [88] allowing for 2 mismatches. Sequences aligning to rRNA were discarded and the remaining reads were aligned to KSHV (GQ994935.1) and human (hg19) genomes. For normalization, uniquely mapped reads were used to calculate the mRNA and ribosome footprint reads per kilobase per million (rpkM), and regions containing multi-mapped reads were masked out. The rpkM/gene for mRNA-Seq and Ribo-Seq were visualized using TreeView (1.1.6).

The sequences that did not align to the viral and host genomes were analyzed with TopHat and HMMSplicer for splice junction discovery using default options [22], [23]. We annotated splice junctions present in at least two time points, with an HMMSplicer score>900 and a TopHat score>7. We determined these thresholds based on previously characterized splice junctions. Two previously reported and two novel splice junctions had low or no TopHat score, but were included in the annotations due to their detection in multiple samples and high HMMSplicer score (ORFK1/ORF4, ORF70, ORF46/47, and Kaposin).

While other strong and reliable bioinformatics tools, such as Cufflinks and Scripture, are available for transcript reconstruction, we were not able to use them for viral mRNAs annotation. These tools are optimized for the analysis of transcripts in genomes where transcriptional units are well spaced and well defined. In the case of the compact KSHV genome, most transcripts use common regulatory features, are overlapping, or are very close to each other. Furthermore, and as shown in our study and previous studies by our lab and others, virtually the entire viral genome is transcribed late during the lytic cycle [6], [9]. These conditions make it difficult for these bioinformatics tools to parse out, identify and predict viral transcripts.

### Prediction of translation initiation sites

The identification of translation initiation sites was done using a machine learning approach as previously described Stern-Ginossar et al., 2012. In total our approach successfully predicted the translation initiation site of 64% (56 of 87) of the previously annotated ORFs (Tables 1, 2 and 3). The sites that were not predicted correspond to regions of low read coverage or overlapping ORFs.

### Annotation of 3′ termini

Stretches of 5 or more consecutive adenosines (polyA), allowing one non-A base for every 5, were removed from the 3′ ends of mRNA sequencing reads before alignment. These reads were aligned to the viral genome and the 3′ end was determined as the last nucleotide before the start of the polyA stretch. To prevent false positives, polyA sequences were only used as evidence of 3′ termini when they mismatched the underlying reference genome sequence.

### Validation of ORF57 splice junction

For the validation of the second splicing event within the 3′ end of the ORF57 transcript we prepared cDNA from 1 ug of total RNA using the qScript cDNA-Supermix (Quanta cat. 95048-025) or SuperScript III First Strand Synthesis System for RT-PCR (Invitrogen cat. 18080-051) with a combination of oligo(dT) and random hexamers, following manufacturers recommendations. PCR was done using as a template 1% of the resulting cDNA. The following primers flanking the 5′ and 3′ ends of the second splice junction were used for 30 cycles of amplification (Fwd: 5′ GGCAAAGACGACGAACTCAT 3′ Rev: 5′ GAGAAGAGACCACGCCTGACT 3′). The resulting products were separated in a 1.2% agarose gel and stained with ethidium bromide for visualization.

For the validation of the 3′ end extension of ORF57, PCR was done as described above using the following primers (A-Fwd: 5′ GGGTGGTTTGATGAGAAGGA, B-Fwd: 5′ TGGCAGAGTGTCTCCCGTAT, C-Rev: 5′ GAGAAGAGACCACGCCTGACT, D-Rev: 5′ ATAATGCCGAAGCCGTTATG)

### Validation of mRNA editing by Sanger sequencing

Total RNA was extracted from cells at different times following induction of reactivation by doxycycline treatment, and cDNA was prepared as described above. Gene specific amplification was done using Phusion High-Fidelity DNA Polymerase (NEB cat. M0530S) for 32 cycles following manufacturer guidelines. The following primers were used for amplification: RTA-74281: Fwd-T7 5′ taatacgactcactatagggACGCGCTGTTGTCCAGTATT 3′, Rev-T3 5′ aattaaccctcactaaagggGTACAGTGTGCCGGACTCCT 3′; RTA-72795: Fwd-T7 5′ taatacgactcactatagggCCTCTCGAATGAGGACCAAA 3′, Rev-T3 5′ aattaaccctcactaaagggGTAGACCGGTTGGAAAACCA 3′; Kaposin-117809: Fwd-T7 5′ taatacgactcactatagggGTTGCAACTCGTGTCCTGAA 3′, Rev-T3 5′ aattaaccctcactaaagggAGGCTTAACGGTGTTTGTGG 3′ ; ORF6-6144: Fwd-T7 5′ taatacgactcactatagggGGGATACTTCTCGGGGAGAG 3′, Rev-T3 5′ aattaaccctcactaaagggGGCCCACTGTGCTCAGTAAT 3′; ORF63-102377: Fwd-T7 5′ taatacgactcactatagggGTTGGAAAATATCGCGTGCT 3′, Rev-T3 5′ aattaaccctcactaaagggTTGTGTGTTCGGTCCTGTGT 3′; ORF59-96434 Fwd-T7 5′ taatacgactcactatagggGGACGTGACCCTCCTGTCTA 3′, Rev-T3 5′ aattaaccctcactaaagggTAACGTCTCCACTGCCTTCC3′.

### Northern blotting

Total RNA was extracted from cells using RNABee (AMSBIO cat. Cs-104B) following manufacturer instructions. Northern blotting was done for 10 ug of RNA, or 100 ng of mRNA per lane using the Ambion NorthernMax system (Invitrogen cat. AM1940). Gene and sense specific riboprobes were synthesized from PCR products using the Ambion MAXIscript T7-T3 Transcription Kit (Ambion cat. AM1326) according to manufacturer guidelines. The primers used for the PCR amplification of gene specific probes are: Probe A (24151–25437): Fwd-T7 5′ taatacgactcactatagggagaCAGTCACAAGCACACAACCC 3′, Rev-T3 5′ aattaaccctcactaaagggagaTTCGGGTGATTAAGCAAAGG 3′; Probe B (25437–25929) : Fwd-T7 taatacgactcactatagggagaCCTTTGCTTAATCACCCGAA, Rev-T3 5′ aattaaccctcactaaagggagaGGTGACCGTACTGCCATACC 3′; Probe C (123441–122984): Fwd-T7 5′ taatacgactcactatagggCGCTAACAGGGGAAACGTTAACCTGC 3′, Rev-T3 5′ aattaaccctcactaaagggCTCATTGCCCGCCTCTATTA 3′

### Mochiview files

File S2 contains the updated KSHV 2.0 annotations, mRNA profiles, and ribosome occupancy (CHX) plots. The database can be opened using the Mochiview file software [26], free for download at http://johnsonlab.ucsf.edu/sj/mochiview-software/

To open the file, import and activate the database. Restart the program and the new database will be available for viewing. The following files are included: GQ994935 sequence, KSHV2.0 location, mRNA_dox8h_minus, mRNA_dox8h_plus, mRNA_dox24h_minus, mRNA_dox24h_plus, mRNA_dox48h_minus, mRNA_dox48h_plus, mRNA_dox72h_minus, mRNA_dox72h_plus, fp_dox8h_minus, fp_dox8h_plus, fp_dox24h_minus, fp_dox24h_plus, fp_dox48h_minus, fp_dox48h_plus, fp_dox72h_minus, fp_dox72h_plus, fp_harr_dox48h_minus, fp_harr_dox48h_plus, fp_harr_dox72h_minus, fp_harr_dox72h_plus

## Supporting Information

Figure S1 **Lytic reactivation of KSHV is induced by exogenous expression of RTA in iSLK-219 cells.** (A) Immunoblot of latent (LANA) and late lytic (K8.1) products in iSLK-219 cells (B) Quantitative PCR of viral DNA, shows DNA replication starting at 48 hpi and increasing with time.
(TIFF)

Figure S2 **Read length distribution and sequencing-coverage for mRNA-seq and Ribo-seq in iSLK-219.** (A) Length of fragmented, size selected mRNA (40–100 nt) and monosome-protected footprints (∼30 nt) in lysates from cells in the lytic cycle (72 hr). (B) Number of reads for mRNA-seq and Ribo-seq of all samples included in this study. Note that the number of mRNA-seq and Ribo-seq reads that align to the viral cycle increase as the lytic cycle progresses.
(TIF)

Figure S3 **Transcription of viral genes is highly permissive during the lytic cycle.** mRNA-seq (red) and Ribo-seq (CHX blue) profiles for the entire KSHV genome at 8 and 72 hr following Dox induction. Note the change in the read density as the viral lytic cycle progresses. The Y axis (number of reads) was cut at 50 (plus strand) and −50 (minus strand) for ease of visualization.
(TIFF)

Figure S4 **ALT and K1-11-AS are modestly bound by ribosomes.** (A and B) mRNA-seq and Ribo-seq (CHX and Harringtonine) profiles for the lincRNAs K1/11-Antisense (A) and ALT (B) at 72 hr post lytic reactivation. Only the strand of RNA coding for the lincRNA is shown. Solid blue arrows represent transcripts, light blue arrow heads coding regions and red thick arrows represent the lincRNA.
(TIFF)

Figure S5 **The highly abundant viral transcript PAN, codes for three putative small peptides.** (A) Timecourse of ribosome accumulation on the putative peptides within PAN. Notice the difference in the scale of the number of reads (y axis). The double head arrow indicates that the PAN transcript continues after the region shown in this figure (B) Accumulation of releasing ribosomes at the stop codon of PAN1.1 (top panel) and K8.1 (bottom panel). Where indicated the mRNA-seq and Ribo-seq (CHX and no drug) profiles for PAN (8 hr) and K8.1 (48 hr) coding regions are shown. (C) mRNA-seq and Ribo-seq of the ORFK7 and putative PAN peptides at 72 hr post reactivation. The right panel is a zoom in of the start codon of ORFK7. Notice the difference in the number of reads on the y axis. (D and E) Ribosome Release Score (RRS) was calculated as RSS = [(footprint reads coding region/footprint reads 3′UTR)/(mRNA reads coding region/mRNA reads 3′UTR)] using the read values from the 48 and 72 hr timepoints. RRS above 14 have been previously calculated for coding transcripts by Guttman *et al.*, thus strongly supporting the coding capacity of PAN [40].
(TIFF)

Figure S6 **Identification of a novel splice junction within the 3′ end of ORF57.** (A) Presence of a novel splice site within the 3′ of ORF57. The ORF57 sequence from 81886 to 83490 (GQ994935) includes the previously annotated 5′ splice and the novel 3′ splice annotated in this study. The splice donor and acceptor sites are highlighted in italic bold. Underlined is the sequence of the 3′ and 5′ introns. (B) Splicing of the second intron of ORF57 was evaluated by end-point PCR on cDNA from latent and lytic BCBL-1 and iSLK-219 cells, using primers flanking the intron boundaries (black solid lines). (C) The presence of a 3′ extension of the ORF57 transcript was confirmed in lytic iSLK-219 and BCBL-1 cells by end-point PCR, using three combinations of primers (A-forward, B-forward, C-reverse and D-reverse) flanking the annotated polyA cleavage site for this transcript. 28S was used as an internal loading control.
(TIF)

Figure S7 **Higher levels of Kaposin mRNA-editing correlate with an increase in ADAR during the lytic cycle.** (A) PCR amplification followed by Sanger sequencing of the region of Kaposin cDNA where mRNA editing occurs. Highlighted by the red box is the edited position, nucleotide 117809. Note the gradual and dramatic change from A to G as the lytic cycle progresses. (B) Quantification of the results obtained in (A). (C) Changes in the levels of ADAR isoforms (p150, p110 and p80) during the lytic cycle. Actin: Loading control, KbZip: Viral reactivation control, NS: not specific. (D) Fold changes in the mRNA and ribosome occupancy levels for two cellular genes that escape the host shutoff response, interleukin 6 (IL6) and the Hypoxia inducible factor 1 (HIF1), one gene sensitive to host shutoff protein kinase, DNA-activated, catalytic polypeptide (PRKDC) and ADAR. The fold change was calculated as the Log2 of the ratio of rpkM at 48 hr over 0 hr, for the mRNA-seq (left panel) or Ribo-Seq (right panel) samples. The black and grey bars represent two independent biological replicates. Note that the changes in ADAR mRNA and ribosome occupancy are very similar to the host shutoff escapee HIF1.
(TIFF)

Figure S8 **KSHV uses alternative start codons to increase peptide diversity.** mRNA-Seq and Ribo-Seq (CHX, Harringtonine and No drug) profiles for ORF K6 (A) and ORF62 at 72 h post reactivation (B). The blue arrowheads represent the annotated ORF and the red arrowheads represent the ORFs expressed from the alternative start codons. The dashed lines mark the multiple translation initiation start sites.
(TIF)

Figure S9 **uORFs and sORFs populate the viral genome in KSHV.** (A) Percentage of uORFs and sORFs starting from canonical and near cognate start codons. Other codons are ATT, ATC, ATA, TTG and ACG (see Table S7). (B) The ORF50-AS transcript is protected by ribosomes. mRNA-seq and Ribo-seq profiles (CHX and Harringtonine) of the ORF50-AS/ORF49 region. The blue and grey arrows represent transcripts, the blue and grey arrowheads represent the coding regions of ORF49 and ORF50 and the yellow solid line marks the region of ORF50-AS where ribosomes bind. (C) Numerous sORFs are found within the K4.1/K4.2 transcript. mRNA-seq and Ribo-seq (CHX and Harringtonine) for the K4/K4.1/K4.2 region. The blue arrow and arrowhead represent the K4 transcript, coding region and uORF. The red arrow and arrowheads represent the K4.1/K4.2 transcript and the numerous uORFs encoded by this transcript. The grey arrow and arrowhead represents the K4.1 and K4.2 genes previously annotated, not detected in this study.
(TIFF)

Table S1 **Transcription start sites in KSHV.** Annotations for the transcription start sites described in this study. The second column contains the sequence of 50 nt 5′ and 10 nt 3′ from the annotated TSS. The TATA box is in bold, italic font. The third column indicates the distance from the start of the TATA box to the TSS. The eight TATA-less genes are indicated as No TATA.
(DOCX)

Table S2 **PolyA sites in KSHV.** Polyadenylation sites were annotated in the KSHV genome using mRNA-seq. Cleavage sites were determined as the last nucleotide before a stretch of 5 or more consecutive adenosines. The sequence in column 7 corresponds to 30 nt flanking the polyA cleavage site. The AA/UUAA and GU sites are in italic, bold font and the cleavage site(s) are in bold font.
(DOCX)

Table S3 **PAN represent a large percentage of the KSHV mRNA-seq.** The PAN mRNA or ribosome footprint (FP) reads were calculated for the region between 28661 to 29690. The percentage of KSHV reads was calculated using the total number of viral reads for mRNA-seq or Ribo-seq for each timepoint. ^1^^.^ The PAN RNA reads in this latent sample are likely coming from the 0.1% of spontaneous lytic cells or from minor cross-contamination during library preparation. ^2^^.^ Technical replicates.
(DOCX)

Table S4 **Splice junctions in the KSHV transcriptome.** Splice junctions were identified and annotated using TopHat and HMMsplicer. The novel splice junctions identified in this study are in bold font. Donor and acceptor sites are in italic, bold font (column 6). Columns 7 and 8 contain the TopHat and HMM generated scores for two replicates at 48 hr (rep1 and rep2) and one at 72 hr post reactivation. These scores reflect the strength of the alignment (HMM splicer) and the number of reads that mapped to the region (TopHat and HMM splicer).
(DOCX)

Table S5 **mRNA editing in KSHV.** mRNA editing events were predicted in silico and were annotated by position and gene. We include in this table a DNA mutation identified in the GQ994935.1 genome in ORF63 at position 102377 (A-to-C change). Three editing events, Kaposin 117809, RTA 72841 and RTA 72795 have been confirmed by Sanger sequencing.
(DOCX)

Table S6 **Alternative start codon usage and internal ORFs in KSHV.** From our Ribo-seq data we identified five viral ORFs (ORF70, K6, ORF54, ORF62 and Kaposin) with multiple in frame initiation codons where ribosomes accumulate. The size of the predicted protein products originating from the alternative start codons is indicated in the column labeled amino acids. In addition, five viral ORFs (ORF10, ORF11, K3, ORF20 and K8.1) contain internal initiation codons, in or out of frame with the primary ORF, where we detect initiating ribosomes. The size of the predicted products is indicated in the column labeled amino acids (Nuc.: nucleotides, M: Manual annotation, P: Predicted in silico).
(DOCX)

Table S7 **Upstream and small ORFs are widely distributed in KSHV.** The translation boundaries for uORFs and sORFs were predicted by the SVM based on the Ribo-seq data from harringtonine treated cells at 72 h post reactivation. The ORF coordinates were then manually curated. The translation efficiency (TE) for the ORFs larger than 15 amino acids was determined as Footprint-rpkM/mRNA-rpkM. For the calculation we excluded the first 45 nucleotides from the start codon. (M: Manual annotation, P: Predicted in silico, N/A: not available, *: Start codons are ambiguous).
(DOCX)

File S1 **Summary of novel features annotated for KSHV.** The novel genomic features annotated in our study include the ORFs derived from alternative start codons, internal, small and upstream ORFs, and polyadenylation sites. All coordinates are annotated based on the human herpesvirus 8 accession number GQ994935.1.
(XLSX)

File S2 **Mochiview files for updated annotation and mRNA-seq/Ribo-seq profiles.** Supplementary file 1 is a Mochiview file [26] containing the updated KSHV 2.0 annotations, mRNA profiles, and ribosome occupancy (CHX) plots for all of the time points analyzed (0, 8, 24, 48, 72 hr). Ribosome occupancy plots for Harr-treated cells are included for the 48 and 72 hr time points. See materials and methods for uploading instructions.
(ZIP)
