# Supplementary figures and images for: KSHV 2.0: A Comprehensive Annotation of the Kaposi's Sarcoma-Associated Herpesvirus Genome Using Next-Generation Sequencing Reveals Novel Genomic and Functional Features
Source: PLoS Pathog. 2014 Jan 16;10(1):e1003847. doi: 10.1371/journal.ppat.1003847 (PMC3894221; doi:10.1371/journal.ppat.1003847)

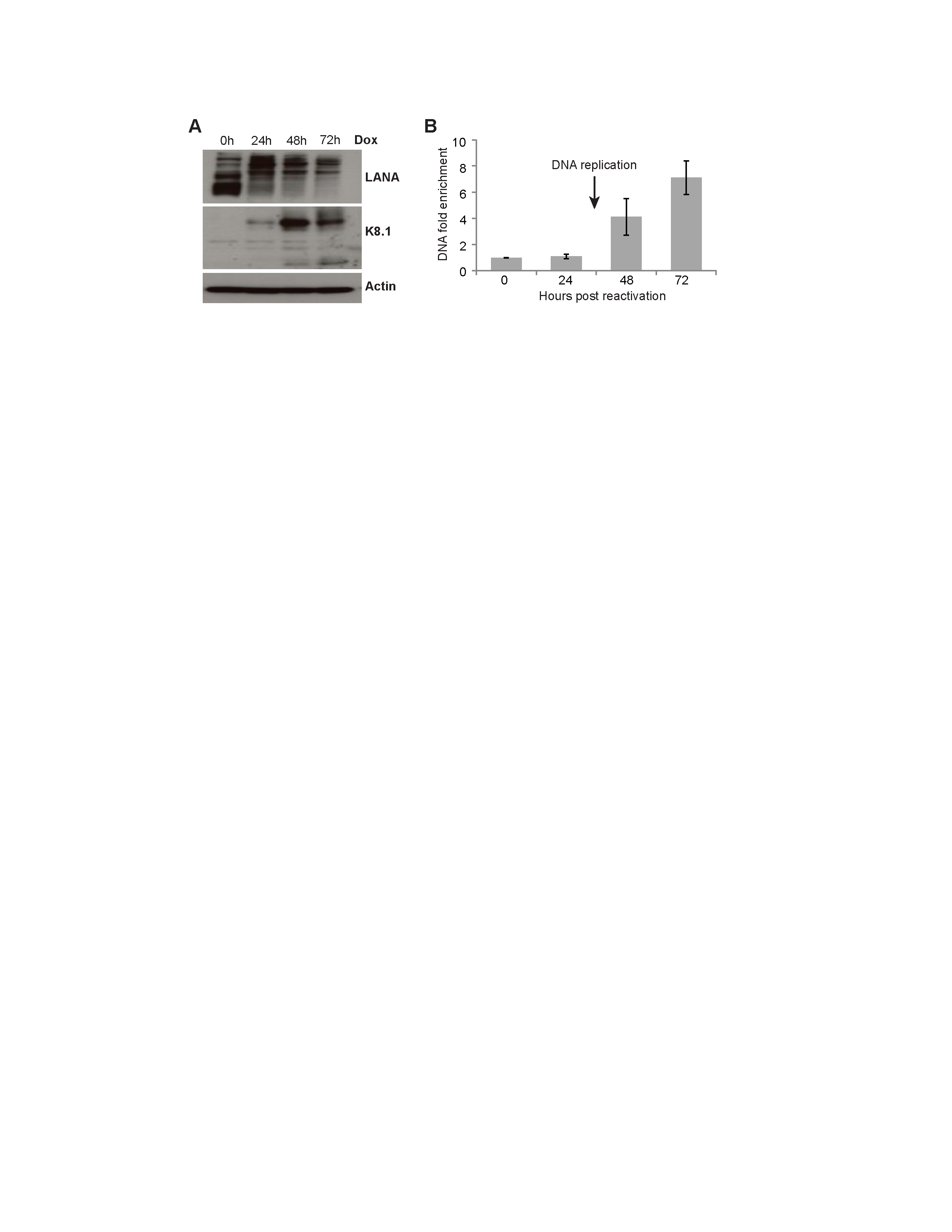

Supplement: Figure S1 — Lytic reactivation of KSHV is induced by exogenous expression of RTA in iSLK-219 cells. (A) Immunoblot of latent (LANA) and late lytic (K8.1) products in iSLK-219 cells (B) Quantitative PCR of viral DNA, shows DNA replication starting at 48 hpi and increasing with time. (TIFF) [file ppat.1003847.s001.tiff]

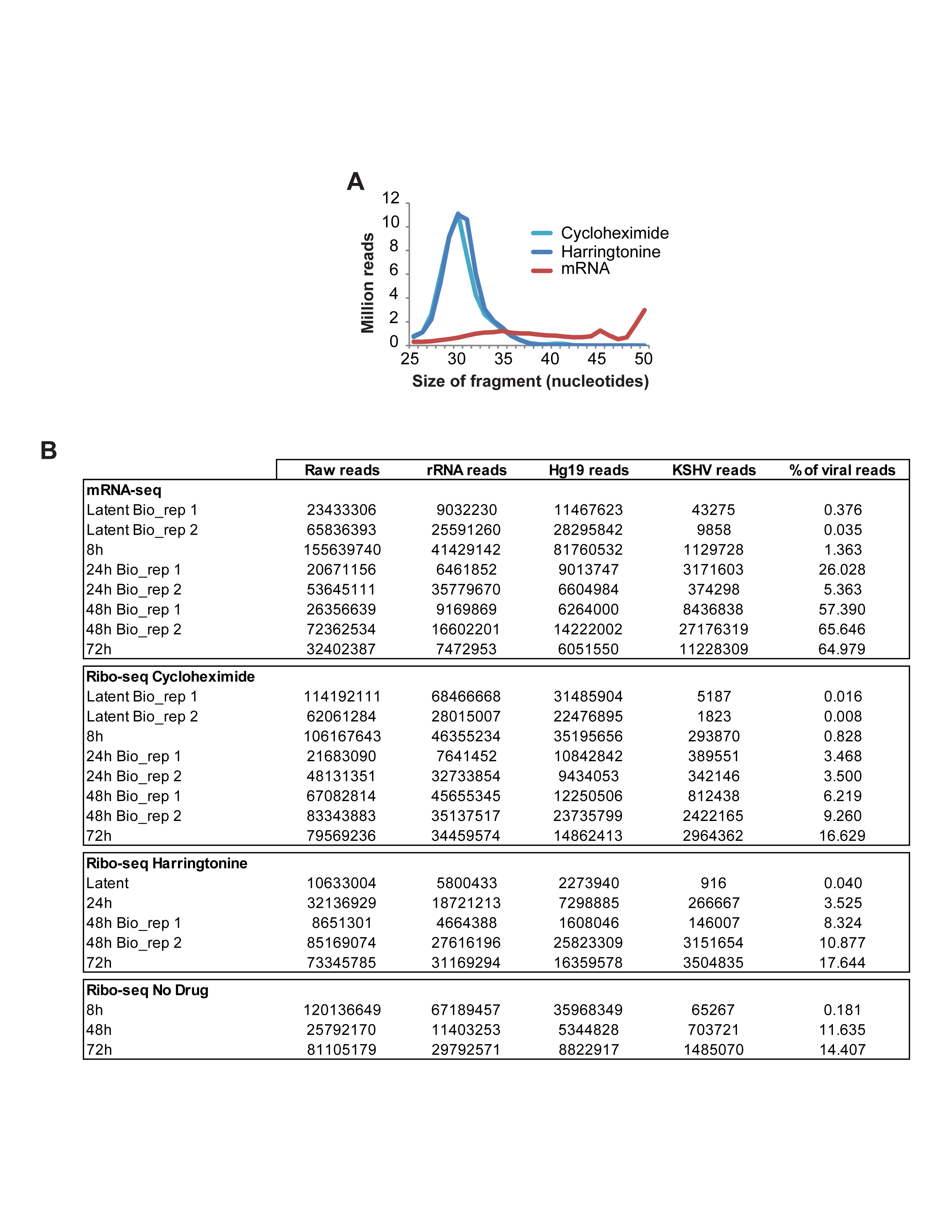

Supplement: Figure S2 — Read length distribution and sequencing-coverage for mRNA-seq and Ribo-seq in iSLK-219. (A) Length of fragmented, size selected mRNA (40–100 nt) and monosome-protected footprints (∼30 nt) in lysates from cells in the lytic cycle (72 hr). (B) Number of reads for mRNA-seq and Ribo-seq of all samples included in this study. Note that the number of mRNA-seq and Ribo-seq reads that align to the viral cycle increase as the lytic cycle progresses. (TIF) [file ppat.1003847.s002.tif]

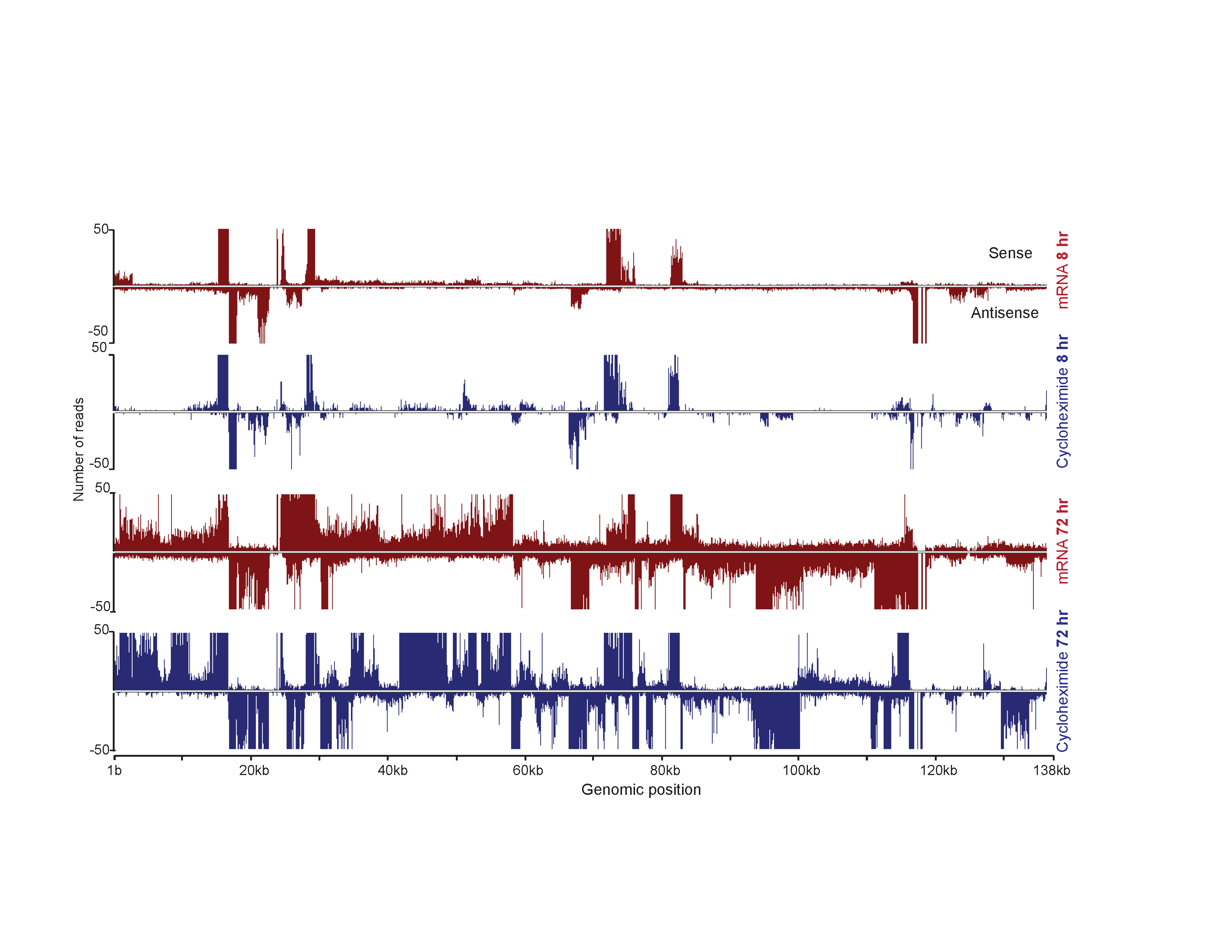

Supplement: Figure S3 — Transcription of viral genes is highly permissive during the lytic cycle. mRNA-seq (red) and Ribo-seq (CHX blue) profiles for the entire KSHV genome at 8 and 72 hr following Dox induction. Note the change in the read density as the viral lytic cycle progresses. The Y axis (number of reads) was cut at 50 (plus strand) and −50 (minus strand) for ease of visualization. (TIFF) [file ppat.1003847.s003.tiff]

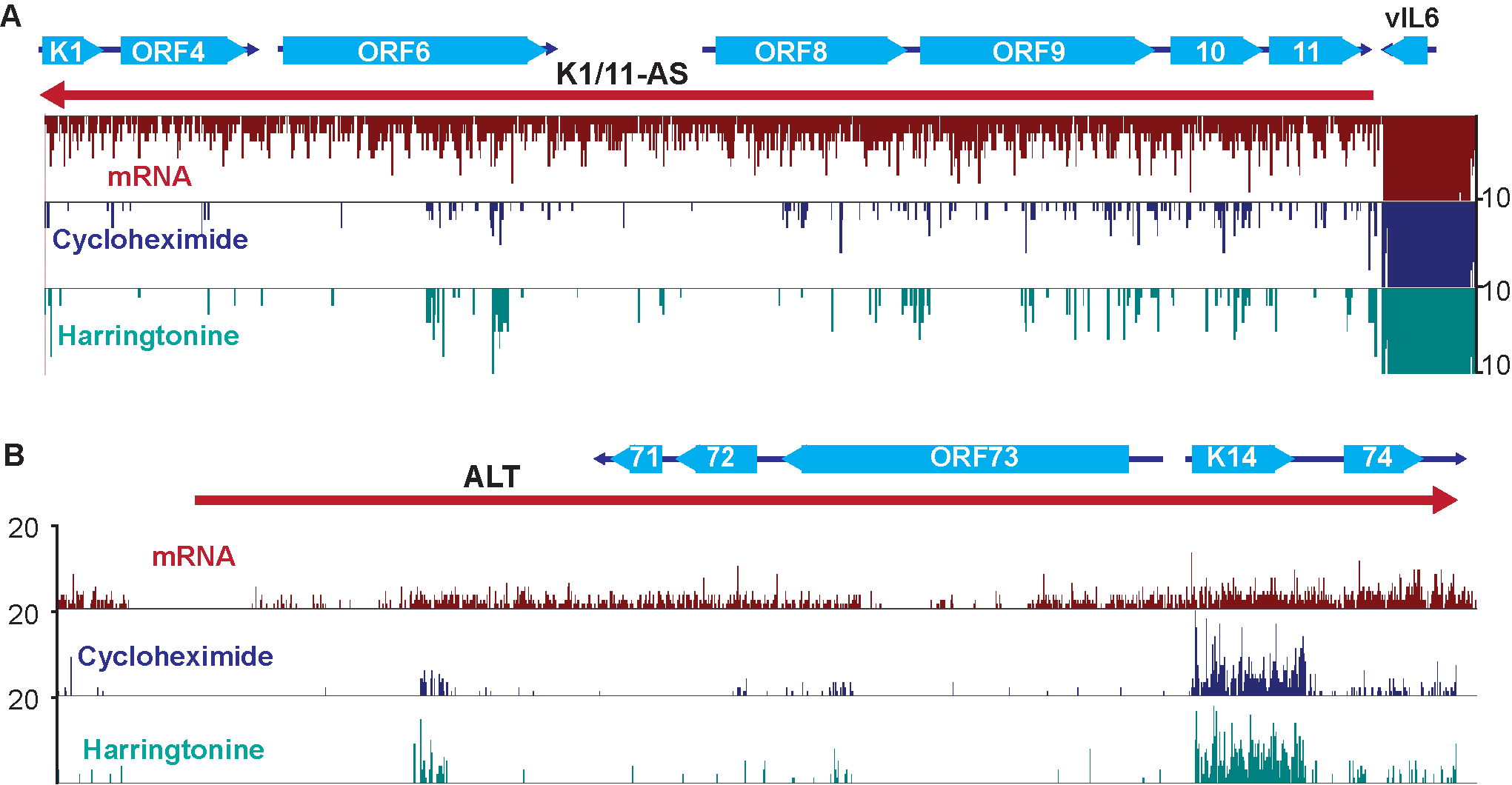

Supplement: Figure S4 — ALT and K1-11-AS are modestly bound by ribosomes. (A and B) mRNA-seq and Ribo-seq (CHX and Harringtonine) profiles for the lincRNAs K1/11-Antisense (A) and ALT (B) at 72 hr post lytic reactivation. Only the strand of RNA coding for the lincRNA is shown. Solid blue arrows represent transcripts, light blue arrow heads coding regions and red thick arrows represent the lincRNA. (TIFF) [file ppat.1003847.s004.tiff]

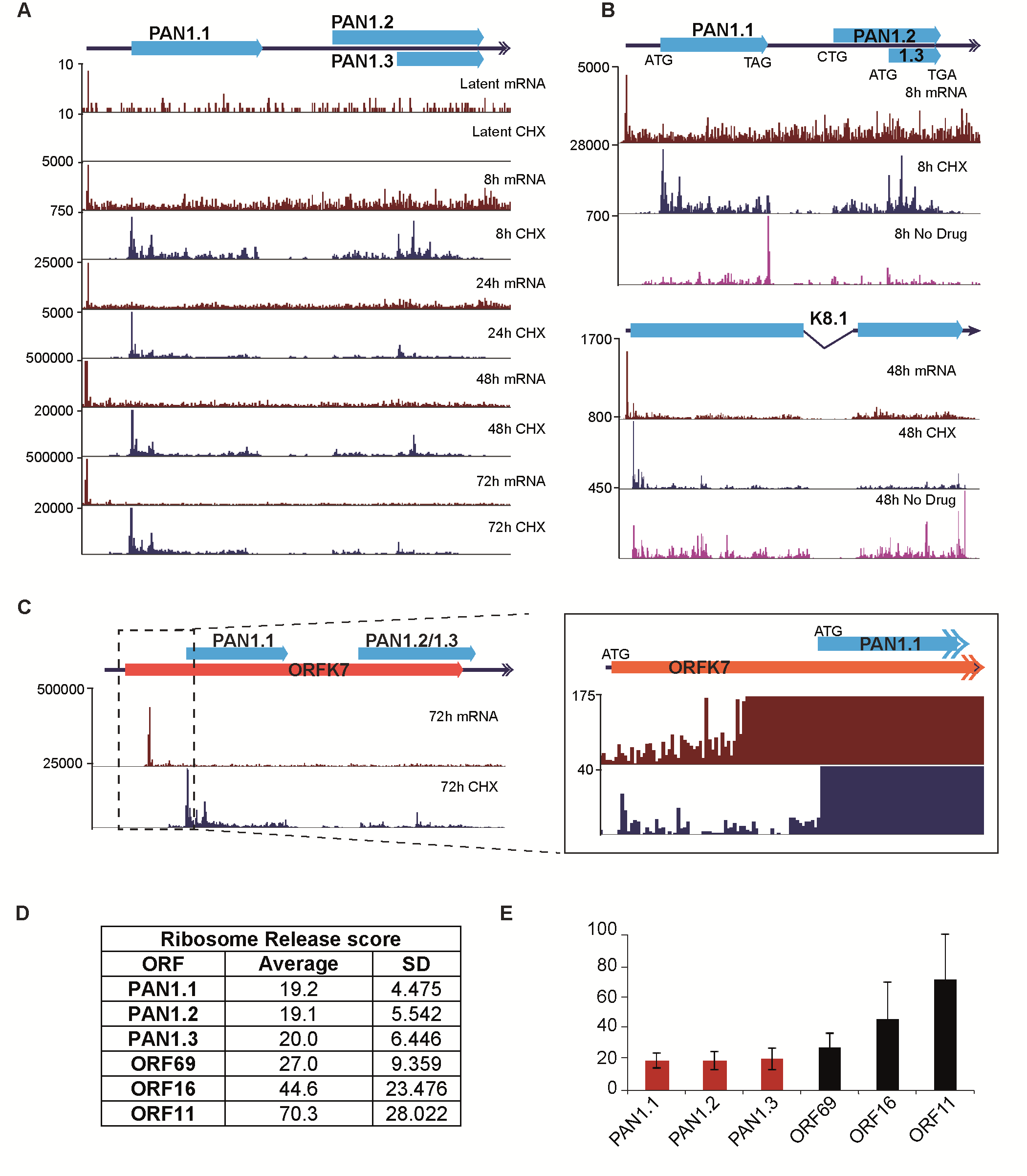

Supplement: Figure S5 — The highly abundant viral transcript PAN, codes for three putative small peptides. (A) Timecourse of ribosome accumulation on the putative peptides within PAN. Notice the difference in the scale of the number of reads (y axis). The double head arrow indicates that the PAN transcript continues after the region shown in this figure (B) Accumulation of releasing ribosomes at the stop codon of PAN1.1 (top panel) and K8.1 (bottom panel). Where indicated the mRNA-seq and Ribo-seq (CHX and no drug) profiles for PAN (8 hr) and K8.1 (48 hr) coding regions are shown. (C) mRNA-seq and Ribo-seq of the ORFK7 and putative PAN peptides at 72 hr post reactivation. The right panel is a zoom in of the start codon of ORFK7. Notice the difference in the number of reads on the y axis. (D and E) Ribosome Release Score (RRS) was calculated as RSS = [(footprint reads coding region/footprint reads 3′UTR)/(mRNA reads coding region/mRNA reads 3′UTR)] using the read values from the 48 and 72 hr timepoints. RRS above 14 have been previously calculated for coding transcripts by Guttman et al., thus strongly supporting the coding capacity of PAN [40]. (TIFF) [file ppat.1003847.s005.tiff]

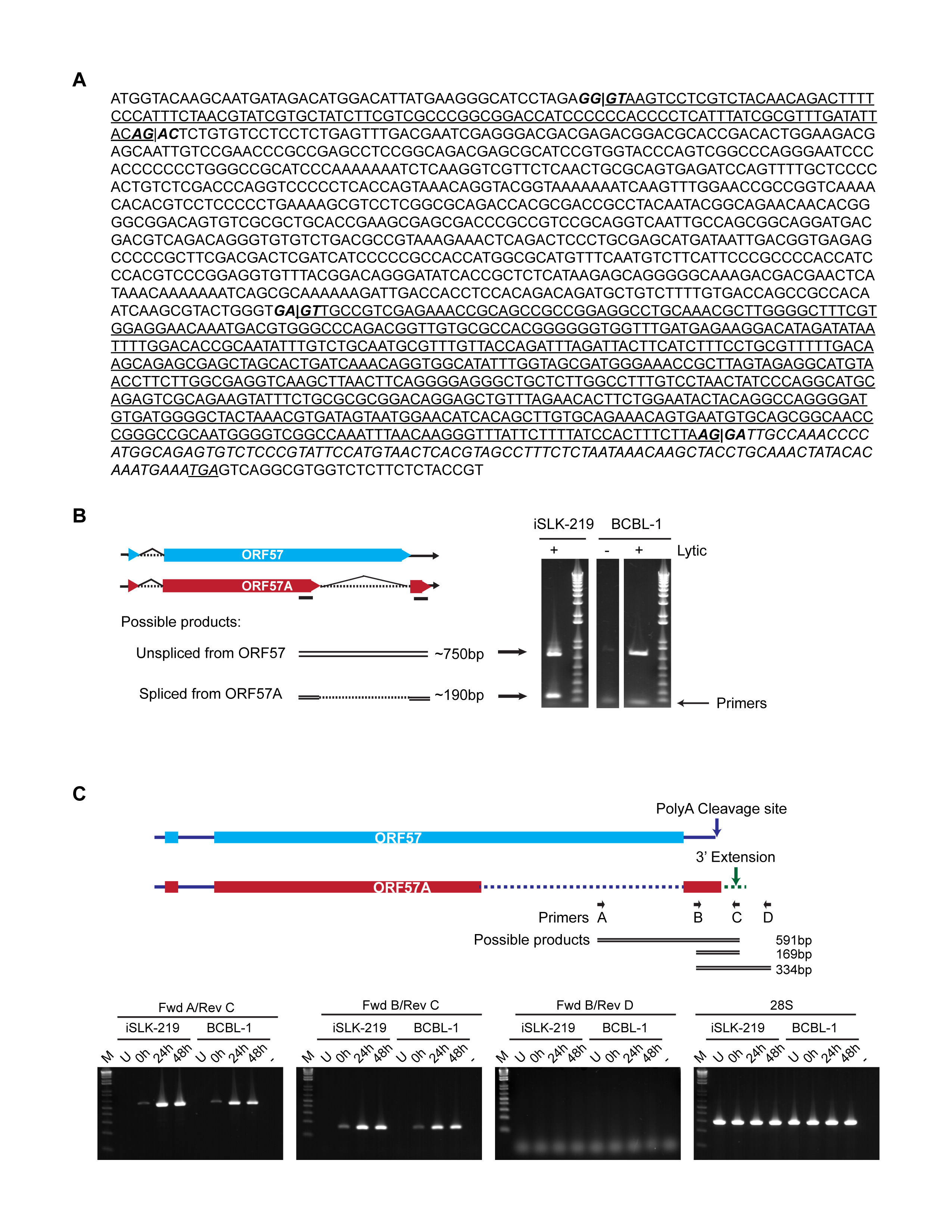

Supplement: Figure S6 — Identification of a novel splice junction within the 3′ end of ORF57. (A) Presence of a novel splice site within the 3′ of ORF57. The ORF57 sequence from 81886 to 83490 (GQ994935) includes the previously annotated 5′ splice and the novel 3′ splice annotated in this study. The splice donor and acceptor sites are highlighted in italic bold. Underlined is the sequence of the 3′ and 5′ introns. (B) Splicing of the second intron of ORF57 was evaluated by end-point PCR on cDNA from latent and lytic BCBL-1 and iSLK-219 cells, using primers flanking the intron boundaries (black solid lines). (C) The presence of a 3′ extension of the ORF57 transcript was confirmed in lytic iSLK-219 and BCBL-1 cells by end-point PCR, using three combinations of primers (A-forward, B-forward, C-reverse and D-reverse) flanking the annotated polyA cleavage site for this transcript. 28S was used as an internal loading control. (TIF) [file ppat.1003847.s006.tif]

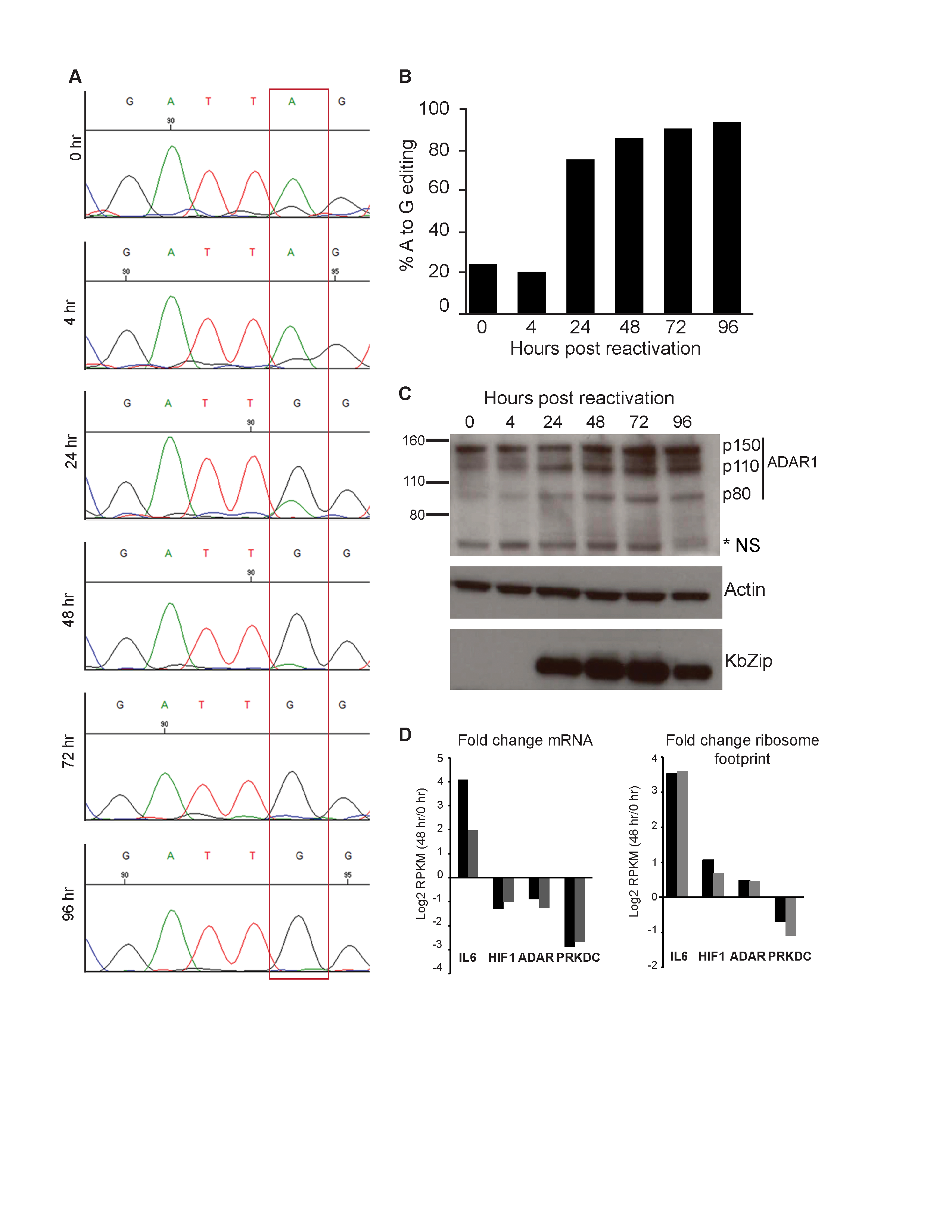

Supplement: Figure S7 — Higher levels of Kaposin mRNA-editing correlate with an increase in ADAR during the lytic cycle. (A) PCR amplification followed by Sanger sequencing of the region of Kaposin cDNA where mRNA editing occurs. Highlighted by the red box is the edited position, nucleotide 117809. Note the gradual and dramatic change from A to G as the lytic cycle progresses. (B) Quantification of the results obtained in (A). (C) Changes in the levels of ADAR isoforms (p150, p110 and p80) during the lytic cycle. Actin: Loading control, KbZip: Viral reactivation control, NS: not specific. (D) Fold changes in the mRNA and ribosome occupancy levels for two cellular genes that escape the host shutoff response, interleukin 6 (IL6) and the Hypoxia inducible factor 1 (HIF1), one gene sensitive to host shutoff protein kinase, DNA-activated, catalytic polypeptide (PRKDC) and ADAR. The fold change was calculated as the Log2 of the ratio of rpkM at 48 hr over 0 hr, for the mRNA-seq (left panel) or Ribo-Seq (right panel) samples. The black and grey bars represent two independent biological replicates. Note that the changes in ADAR mRNA and ribosome occupancy are very similar to the host shutoff escapee HIF1. (TIFF) [file ppat.1003847.s007.tiff]

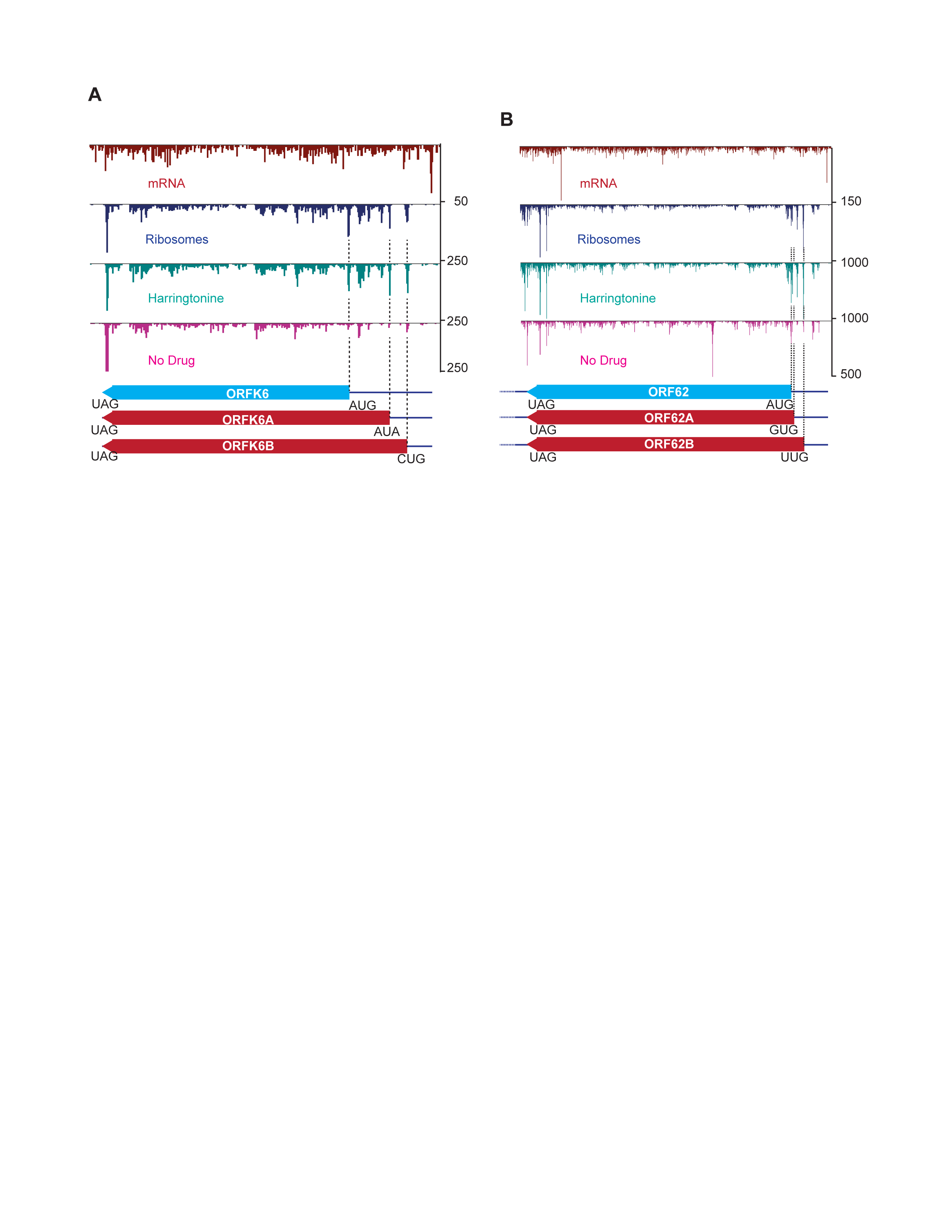

Supplement: Figure S8 — KSHV uses alternative start codons to increase peptide diversity. mRNA-Seq and Ribo-Seq (CHX, Harringtonine and No drug) profiles for ORF K6 (A) and ORF62 at 72 h post reactivation (B). The blue arrowheads represent the annotated ORF and the red arrowheads represent the ORFs expressed from the alternative start codons. The dashed lines mark the multiple translation initiation start sites. (TIF) [file ppat.1003847.s008.tif]

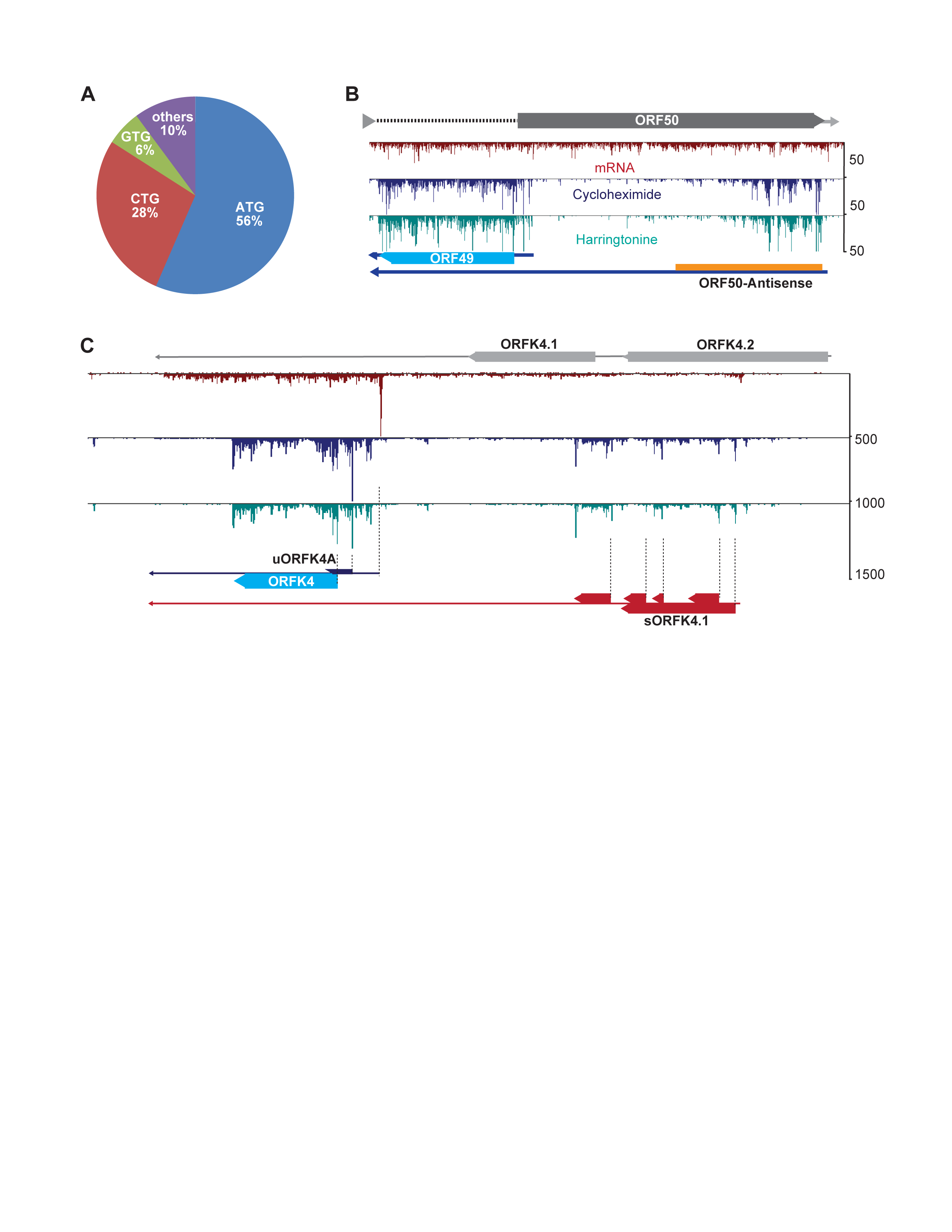

Supplement: Figure S9 — uORFs and sORFs populate the viral genome in KSHV. (A) Percentage of uORFs and sORFs starting from canonical and near cognate start codons. Other codons are ATT, ATC, ATA, TTG and ACG (see Table S7). (B) The ORF50-AS transcript is protected by ribosomes. mRNA-seq and Ribo-seq profiles (CHX and Harringtonine) of the ORF50-AS/ORF49 region. The blue and grey arrows represent transcripts, the blue and grey arrowheads represent the coding regions of ORF49 and ORF50 and the yellow solid line marks the region of ORF50-AS where ribosomes bind. (C) Numerous sORFs are found within the K4.1/K4.2 transcript. mRNA-seq and Ribo-seq (CHX and Harringtonine) for the K4/K4.1/K4.2 region. The blue arrow and arrowhead represent the K4 transcript, coding region and uORF. The red arrow and arrowheads represent the K4.1/K4.2 transcript and the numerous uORFs encoded by this transcript. The grey arrow and arrowhead represents the K4.1 and K4.2 genes previously annotated, not detected in this study. (TIFF) [file ppat.1003847.s009.tiff]
