# Supplementary material for: KSHV 2.0: A Comprehensive Annotation of the Kaposi's Sarcoma-Associated Herpesvirus Genome Using Next-Generation Sequencing Reveals Novel Genomic and Functional Features
Source: PLoS Pathog. 2014 Jan 16;10(1):e1003847. doi: 10.1371/journal.ppat.1003847 (PMC3894221; doi:10.1371/journal.ppat.1003847)
Supplement: Table S1 — Transcription start sites in KSHV. Annotations for the transcription start sites described in this study. The second column contains the sequence of 50 nt 5′ and 10 nt 3′ from the annotated TSS. The TATA box is in bold, italic font. The third column indicates the distance from the start of the TATA box to the TSS. The eight TATA-less genes are indicated as No TATA. (DOCX) [file ppat.1003847.s010.docx]

**Table S1. Transcription start sites in KSHV**

| **Gene** | **Sequence** | **Distance TATA to TSS** |
| --- | --- | --- |
| K1 | ***TACTAA***TTTCAAAGGCGGGGTTCTGCCAGGCATAGTCTTTTTTTCTG | 36bp |
| ORF4 | TTTTATGAGGTCGGATACT***TATTAAA***AGCATTGTCTTAAGTACATTAAAAGGACATTGTAT | 31bp |
| ORF6 | GGCAGAAAATTCACACCG***TATATAAA***CAAGGAAAGGGGACTCTGCGCGCTTAAGCGCCAAG | 31bp |
| ORF8 | TAAAAAATATGGCTGGA***TATTTAA***AGACCTGTACGCCCTTCTGTACCACCACCTGCAATTG | 32bp |
| ORF11 | GGCGTGGCAATGGCTTGCTATATCCACCCCGGTAAGGCAGCCAGCCAGGCACCATACAGCT | No TATA |
| vIL6 | GGCGCTCACTGGCCCCTGGCCAGTTAGGC***TATTTTTA***ACCCGGGTTTTACATGACTTTGCG | 20bp |
| ORF70-K3 | TTTATAGAAGAGCCTGAGTATACATGTAGGTCTCCGAGCACTGCGAGTTGATGGGTGTCTC | No TATA |
| K3_internal | GCTGCGACGTGGGTTTGGC***TATATATA***GTGGGCGGTCATGACCCAGAGATAGATCACGTCG | 30bp |
| ORFK4 | TTGGGGTGTTGGCCTTCG***TAATAAA***AGCCCAGGATCCTGGAGTTTACAGGTAGGCGTCGTA | 31bp |
| ORFK4.1/4.2 | CTGGGATAGCTTGGAG***TATTAAA***CCTGGGAACTATGATCCTTTAATCCCATTTGAGACTTT | 33bp |
| 1.4Kb | GTTACACCCCTTCGCCGGGAACGC***TATAAA***AACGAGGGACAGCAGCCCCCCTCGCGCACTG | 25bp |
| ORFK5 | AGCTGCCTAGAACTAACCAC***TCATAAAA***CCTCCAAACACAGGCGGAGAGCGCGGGGGTGGA | 29bp |
| ORFK6 | TCGCCCGGGTGGCAATGGAAACCTGGCTTAATGACAGAACTCAAAGCCCACAAAGGCGCCA | No TATA |
| ORFK6_2 | TGGCGGTTAGCATACGCCAC***TATAAA***TAAACAGGTGAGACCAGAATCAGGTAAGCATATAA | 29bp |
| ORFK7 | TGTTTTGGGGTTATCTACA***TATTATA***TTCCTTATCCCGACTGGTTGCGGAAGTATTCGCAG | 30bp |
| PAN | AAAATATGGGAACACTGGA***GATAAAA***GGGGCCAGCTTGAGTCAGTTTAGCACTGGGACTGC | 30bp |
| ORF16 | TCGATTGGGATGGGGGTGTGGGATGGGGGTGTGGGATGGGGGTGTGGGATGGGGGTAAATG | No TATA |
| ORF17.5 | GCATATTGAGCCCGGCG***TATTTAA***AGGCCAGCCAATTCCCGGTCGGCATCCAAGCCGTCAC | 32bp |
| ORF17.5_2 | GCCAGCATATTGAGCCCGGCG***TATTTAAA***GGCCAGCCAATTCCCGGTCGGCATCCAAGCCG | 28bp |
| ORF17 | GCTCTTGGGCGTGGAATG***TATTTAAA***TTCTTATTTTGCAAAAAGCGCCACATGAGCCTCCT | 31bp |
| ORF21 | ATAGATCGGGCAGGGTGGAGTACTTGAGGAGCCGGCGGTAGGTGGCCAGGTGGGCCCGGTT | No TATA |
| ORF25 | AAAAGAGTCCCTTCTTGATG***TATAAAA***GGGTGGAGGCGTTCCCCCAGGAGTAGTCTGCGTA | 29bp |
| ORF26 | GTGGCGCCGATGAAGAGAC***TATTAAA***GCTCGGAAACAAGGTGGTGTATTAGCTAACCCTTC | 30bp |
| ORF27 | ATCTTCAGAGTCTCAGTTC***TATATTTAA***TCTTGGCCCCAGACTGCACGTGTATGCCTACTC | 30bp |
| ORF28 | ATTGCTCTGTTGTAAAC***TATATATAA***GTTAAACCAAAATTCGCAGGGAGACAAGGTGACGG | 32bp |
| ORF33 | TGTTACCTGGGGGTTTTGC***TATTAAA***GGCCGCTATAGGGCGTCGAAGGAGGATCTGGTGTT | 30bp |
| ORF34 | TTTATTCTGTGCACCACGC***CAATAAA***AGGGTGCGCCATCCGTGCCGTTTTGGGACAGTGTC | 29bp |
| ORF35 | TTGAGTGGAAAAGGGACCCAG***TATATAA***CAGGCAATGTTCAGACCCAAAGGTGTCCAACTA | 28bp |
| ORF38 | TACTCGGGGTCAAATCAG***TATTAAA***GCCCGCTTCAAAGCCAACCTCTTCGTGAACGTCCGT | 31bp |
| ORF39 | GCTTCTGCCCGGGGCGCG***TATTTAA***AAGTCAACCGCGATAGGCAGTGGCATCAGATTGGCT | 31bp |
| ORF45 | GATTGGTTCACGAGTTTCATCATCAGTAGCATATCCTCAAAGTTAGAACATTGCGTTTTTC | No TATA |
| ORF45.1 | CTGCTGCGATCCAGAGGA***TATTAAA***GTGGTGATCTTAGGCCAGGACCCGTACCACAAGGGC | 32bp |
| ORF46-47 | GCATGCGTGAACCCTCAC***TATATAAA***ACAGATATTACGGAAGGGGGCTCCCTGGGGAGCAC | 31bp |
| ORF50 | GGTGGCAATGACACGTCCCC***TTTAAAAA***GTCAACCTTACTCCGCAAGGGGTAGTCTGTTGT | 29bp |
| ORFK8 | GGTATTTCCTCCGTTGTCGACTATAACCTGGCGTGTAAACGTGTAACCCTGCCAAATGCCC | No TATA |
| ORFK8.1 | TGTTATCCGGCAGCAA***TATTAAA***GGGACCGAAGTTAATCCCTTAATCCTCTGGGATTAATA | 33bp |
| ORF52 | GCTATGGTTTTTCAATAAAG***TATTTAAA***GCTGGGTATAAAGGAGGGATTTGGGCTTTTGTT | 29bp |
| ORF53 | TCGGACTGCATTGCAGAGTG***TATATAA***GAGGCGAACGCGGAACGTTGGATAGACGGCTTGG | 29bp |
| ORF55 | GGTCGCTGAGCAGCA***TATATTTAA***TGTCGGACGCCTCCAGTGGCCGGCCGCCAAGGAACCC | 34bp |
| ORF57 | GGACTGGCCAGTTAATCCCAC***TATATAA***CCTGGCTGCCAGGTTCCCAAAATAGCCCGCGGC | 28bp |
| ORF58-59 | CACCTCCCCTAAAAGTTC***TATTTAA***GGCAGTCTATCGTCTCCAGAACACCCAGTGTGCGCG | 31bp |
| ORF60-61 | TCAGCTGAGGCACGAGCTG***TATATAAA***TCGGCTTGTTTTTGGGGCGGTTTGTACGCTGGGC | 30bp |
| ORF62 | CGGCAGTTGGGCAACAG***TATTTAAAA***ACTGCTCTGTTGTGCTGCTGTTTAAGAGGGCAGGC | 32bp |
| ORF65 | GACTGCGAGGCTGCCC***TATTAAA***GCACCGTGACGTGCGCGGACTGCTTCAGCTCACCTCAC | 33bp |
| ORF69 | GCCTGCCCAGGGAGCAGGAG***TATAAAA***AGCTCAGGGCATCCGCGGCCGCCGGACAGCTCCT | 29bp |
| ORFK12 | CCCCCTTCGCAGGAAACGC***TATAAA***GAAGAGGGGAGACCGACCCCCCTCGCGCATTACGCG | 30bp |
| ORF71-72 | TCCACAGGAAATGA***CATAAAA***GCCACACCTCTCCCCCTTTTTCCTCCCTAGAAGCCACCGT | 34bp |
| ORF73 | AACTCCGCCCTCCACTACGCGGCGCCCGGGA***CAATCAAA***AAATGTGTGTATCATTTGGAGG | 17bp |
| ORFK14/74 | GGGCGGGCGCTACTCACTG***TTTATAA***GTCAGCCGGACCAAGCTGCTGCTCTTGGGGACGTG | 30bp |
| ORF75 | CGATACCTTTTTTTGAG***TATTTGA***GGTTAGTGACATGGCTACATGTAACTGTGGATTCCAC | 32bp |

The second column contains the sequence of 50nt 5’ and 10nt 3’ from the annotated TSS. The TATA box is in bold, italic font. The third column indicates the distance from the start of the TATA box to the TSS.
