# Supplementary material for: KSHV 2.0: A Comprehensive Annotation of the Kaposi's Sarcoma-Associated Herpesvirus Genome Using Next-Generation Sequencing Reveals Novel Genomic and Functional Features
Source: PLoS Pathog. 2014 Jan 16;10(1):e1003847. doi: 10.1371/journal.ppat.1003847 (PMC3894221; doi:10.1371/journal.ppat.1003847)
Supplement: Table S2 — PolyA sites in KSHV. Polyadenylation sites were annotated in the KSHV genome using mRNA-seq. Cleavage sites were determined as the last nucleotide before a stretch of 5 or more consecutive adenosines. The sequence in column 7 corresponds to 30 nt flanking the polyA cleavage site. The AA/UUAA and GU sites are in italic, bold font and the cleavage site(s) are in bold font. (DOCX) [file ppat.1003847.s011.docx]

**Table S2. PolyA sites in KSHV**

| **Gene(s)** | **Position** | **Strand** | **Sites** | **Sequence** |
| --- | --- | --- | --- | --- |
| K1/ORF4 | 2956 | + | 1 | agataccgacc***aatacaa***gacaactaatat**TA**accata***gtgtgcgtttctttg***tataaaat |
| ORF6 | 7013 | + | 2 | gtcact***aataaa***cgacgttgaaattt**TC**tt**TA**aacgtc***ttagtagcgtttttt***attgtata |
| ORF8/9/10/11 | 17054 | + | 2 | ttattgctttcaa***ataaaa***cggtgttctct**CA**acct**CCT**cc***gggctcactagtattgtgtt*** |
| vIL6 | 17163 | - | 3 | tctagac***aataaa***gattaactgtcta**CA**gt**CA**c**TA**tgtc***tttgtgtgtgttatgtattagg*** |
| ORF70/K3 | 18575 | - | 2 | cccttatgtttc***attaaa***agtatataagag**CACA**ct***gtgtggttggtggtgttt***acctata |
| K4/K4.1-4.2 | 21274 | - | 3 | tcttgtaaa***aataaa***tgtgaatgtcaga**CACACA**c***tttttgttcttgtgatgttttctggt*** |
| 1.4Kb_upst * | 25069 | + | 3 | tcgttatccaatatc***attaaa***aaccacacc**GA**aa**TTTA**caca***ggtagcacgtcaccgtgtt*** |
| 1.4Kb | 25394 | + | 6 | aggctcttga***aataaa***caatgtttacc**GA**g**TA**aaaggttccactcaccctca***tttgtcgtt*** |
| K5 | 25501 | - | 2 | cctc***attaaa***aacaagtagctgttgacttt**CA**aa**CA**accc***gcgtgcgatatgttttc***catc |
| K6 | 26843 | - | 2 | tcc***aataaa***gctgttgtggtttttctct**CATA**acta***gtgatgacgcgtgtcccttattt***ca |
| PAN | 29690 | + | 5 | tacaacata***aataaa***ggtcaatgtt**TA**at**CATATTT**ctgac***ttgtgtcttgacttgcgt*** |
| ORF17/17.5 | 30563 | - | 5 | tttgaaaaaaa***aataaa***catacgttttgaa**CAATGTTCA**tttaat***gtttatgttgtttgt***a |
| ORF16 | 30566 | + | 1 | aaacaacataaac***attaaa***tgaacattgtt**CA**aaacgtat***gtttattttttttt***caaacag |
| ORF18 | 33273 | + | 1 | tatctc***attaaa***cctactgcctgtcagatt**TA**caaatggtcc***gggttgtttgtggg***acacg |
| ORF21/22 | 39147 | + | 1 | ttattagacggtc***aataaa***gcgtagatttt**TA**aaaggtttcc***tgtgcattctttttgt***atg |
| ORF23/24 | 39048 | - | 1 | cgagaaccc***aataaa***agagagaattagaaa**CA**aagcactggc***tgcgcgtcttctatacatg*** |
| ORF27 | 48586 | + | 4 | ta***aataaa***ggtgtgtcactggt**TA**cacca**CG**at**TA**aaaac**CA**ctcac***tgagatgtcttttt*** |
| ORF30/31/32/33 | 53911 | + | 3 | ca***aataaa***ccgaagtactgct**TA**aa**CA**atc**CA**aacaac***tggtgcgtcttttgtggggcctt*** |
| ORF34/35/36/37/38 | 58691 | + | 2 | agaaaaaa***aataaa***gaagcaatttattaag**CA**aaca**GTA**tg***gttttctgtacgtattttat*** |
| ORF39 | 58701 | - | 2 | ccaccacgg***aataaa***atacgtacagaaaac**CATA**c***tgtttgcttaataaattgcttcttta*** |
| ORF42//43 | 62232 | - | 2 | tttcaa***aataaa***gataaaagcctggtcca**GCC**tc**TA**gct***gtagagtcatttcctttgcgt*** |
| ORF40-41 | 62375 | + | 1 | gatggtatcac***aataaa***aaatgtttactgg**GT**ccgcgca***ggtttgtttgtcatcttcattc*** |
| ORF44 | 67134 | + | 1 | gtttggg***aataaa***gcatgagacttgacacc**TA**taa***tggtctgtattgacaccattctttta*** |
| ORF45/46/47 | 67143 | - | 4 | gata***aataaa***agaatggtgtcaata**CAGACCA*ttataggtgtcaagtctcatgctttatt***c |
| ORF49 | 71432 | - | 1 | tgttcagtatac***aataaa***aaggtcgatctt**TA**ccttgtcatc***ttgcgccatttttgtggct*** |
| ORF52/53 | 76523 | - | 4 | atgtgaac***aataaa***cacgtttattta**CTTTATGG**ttt***tgtgttacactatgtagggtttc*** |
| ORF50/K8/K8.1 | 76554 | + | 4 | aaagta***aataaa***cgtgtttattgtt**CACA**t**GATA**aagagtggtactc***tttactggtttggg*** |
| ORF55 | 78521 | - | 1 | aatcgaacaat***aataaa***aacattggctgt**GTA**cactc***gtttttttatttgggggatgctgt*** |
| ORF54 | 78588 | + | 1 | tggtaccagag***aataaa***gccaacctatgtc**GA**acc**TA**tc***gcgctttctgtcgtctcttcca*** |
| ORF56/57 | 83453 | + | 3 | ct***aataaa***caagctacctgcaaa**CTATACA**caaa***tgaaatgagtcaggcgtggtctcttct*** |
| K9 (vIRF1) | 83605 | - | 1 | tatcatatc***aataaa***gagaccaaaa**CA**ttggc***gggtgtgtcttgtttgttggctccacgt*** |
| K10 | 85823 | - | 1 | agtcca***aataaa***ccagacacgacatttcgc**CA**atta***ttgcaccatttattgtggatacttg*** |
| K10.5 | 89190 | - | 1 | cggaatcgata***aataaa***atagaaagCAtttgagagcgtctc***ggtttcgtatgtcactgtt***a |
| K11 | 91573 | - | 2 | tc***attaaa***ggttagcgccaccgtgtggctgCAaaataaa***gtctgagtggttattttttt***cc |
| ORF58/59/60/61/62 | 94287 | - | 4 | gtcggtaata***aataaa***gttgttggc**TA**at**TG**aa**TA**a**TA**ca***tgtgtttttcttggtttgttg*** |
| ORF65/66/67/67.5 | 116127 | - | 3 | gcgtct***aataaa***actaatcagtgt**TA**t**CT**t**TG**cagtgtcta***tgtctgtgttttgt***aaactg |
| ORF68/69 | 117240 | + | 1 | tgttcaggtggt***aataaa***gtcattaaacga**CA**aa***gtgattcttttaatctgtttattgttt*** |
| Kaposin | 117250 | - | 3 | gttcaaaaac***aataaa***cagat**TA**aaa**GA**at**CA**c***tttgtcgtttaatgacttt***attaccacc |
| Kaposin_upst* | 117688 | - | 1 | gagtgtcagtaa***aataaa***atacaaaagca**CA**atcac***ggttgcaccaagcacaacatt***aaa |
| ORF71/72/73 | 122513 | - | 1 | tgattt***aataaa***cactaacaagttttgtaa**GA**atcattagaa***tgcgtgcttctgtttg***aag |
| ORF75 | 130818 | - | 2 | tttaatcgcat***aataaa***acaaa**TA**catagt**CA**catctgtgtacaaacca***gattcgcctctc*** |
| ORFK14/74 | 130870 | + | 1 | gcg***attaaa***tgaggggtctgatcccaaaag**CA**atgttta**gtggtggtcgttgatcttcttg** |

Polyadenylation sites annotated in the KSHV genome using mRNA-seq. The sequence in column 7 corresponds to 30nt flanking the polyA cleavage site. The AA/UUAA and GU sites are in italic, bold font and the cleavage site(s) are in bold font

*These PAS are upstream of a major site for the 1.4Kb or Kaposin.
