# Supplementary material for: KSHV 2.0: A Comprehensive Annotation of the Kaposi's Sarcoma-Associated Herpesvirus Genome Using Next-Generation Sequencing Reveals Novel Genomic and Functional Features
Source: PLoS Pathog. 2014 Jan 16;10(1):e1003847. doi: 10.1371/journal.ppat.1003847 (PMC3894221; doi:10.1371/journal.ppat.1003847)
Supplement: Table S3 — PAN represent a large percentage of the KSHV mRNA-seq. The PAN mRNA or ribosome footprint (FP) reads were calculated for the region between 28661 to 29690. The percentage of KSHV reads was calculated using the total number of viral reads for mRNA-seq or Ribo-seq for each timepoint. 1 . The PAN RNA reads in this latent sample are likely coming from the 0.1% of spontaneous lytic cells or from minor cross-contamination during library preparation. 2 . Technical replicates. (DOCX) [file ppat.1003847.s012.docx]

**Table S3. PAN represent a large percentage of the KSHV mRNA-seq**

| **Sample** | **PAN mRNA reads** | **% of KSHV reads** | **PAN FP reads** | **% of KSHV reads** |
| --- | --- | --- | --- | --- |
| Uninduced^1^ | 34473 | 79.9 | 280 | 1.24 |
| Uninduced | 595 | 6.1 | 4 | 0.03 |
| 8h | 738675 | 65.5 | 21551 | 0.19 |
| 24h | 2922886 | 92.2 | 64478 | 1.29 |
| 24h^2^ | 293764 | 78.5 | 73457 | 1.53 |
| 48h | 7750239 | 91.9 | 116391 | 1.32 |
| 48h^2^ | 5396949 | 84.8 | 95584 | 1.11 |
| 48h | 24299499 | 89.4 | 295944 | 1.71 |
| 72h | 9323536 | 83.1 | 358019 | 1.42 |

The PAN mRNA or ribosome footprint (FP) reads were calculated for the region between 28661

to 29690. The percentage of KSHV reads was calculated using the total number of viral reads for

mRNA-seq or Ribo-seq for each timepoint.

**^1.^** The PAN RNA reads in this sample are likely coming from the 0.1% of spontaneous lytic cells or from cross-contamination during library preparation.

**^2.^** Technical replicates
