# Supplementary material for: KSHV 2.0: A Comprehensive Annotation of the Kaposi's Sarcoma-Associated Herpesvirus Genome Using Next-Generation Sequencing Reveals Novel Genomic and Functional Features
Source: PLoS Pathog. 2014 Jan 16;10(1):e1003847. doi: 10.1371/journal.ppat.1003847 (PMC3894221; doi:10.1371/journal.ppat.1003847)
Supplement: Table S4 — Splice junctions in the KSHV transcriptome. Splice junctions were identified and annotated using TopHat and HMMsplicer. The novel splice junctions identified in this study are in bold font. Donor and acceptor sites are in italic, bold font (column 6). Columns 7 and 8 contain the TopHat and HMM generated scores for two replicates at 48 hr (rep1 and rep2) and one at 72 hr post reactivation. These scores reflect the strength of the alignment (HMM splicer) and the number of reads that mapped to the region (TopHat and HMM splicer). (DOCX) [file ppat.1003847.s013.docx]

**Table S4. Splice junctions in the KSHV transcriptome**

|  |  |  |  |  |  | **HMMsplicer/TopHat** | | |
| --- | --- | --- | --- | --- | --- | --- | --- | --- |
| **Gene(s)** | **sense** | **Start** | **End** | **Intron size** | **Donor/Acceptor** | **48h_rep1** | **48h_rep2** | **72h** |
| K1/ORF4 | + | 966 | 2609 | 1643 | ctgacattcag/***gt***aa...***ag***/attctgcaga | 1197/8 | N/A | 1208/10 |
| K1/ORF4 | + | 2000 | 2609 | 609 | aaaaccaaatg/***gt***aa...***ag***/attctgcaga | 1354/9 | 1196 | 1294 |
| K1/ORF4 | + | 2234 | 2609 | 375 | gaaagcgaaag/***gt***aa...***ag*/**attctgcaga | 1313/16 | 1227/79 | 1303/34 |
| **ORF70/K3** | **-** | **19560** | **19680** | **120** | agcattacca/***gt***ag...***ag***/gatatggaaga | N/A | 844 | 1331/19 |
| **ORF70/K3** | **-** | **19560** | **19702** | **142** | ggcttgtaca/***gt***ga...***ag***/gatatggaaga | N/A | 1069/32 | 1099/11 |
| ORF70/K3 | - | 19560 | 20034 | 474 | agtatagcag/***gt***ag...***ag***/gatatggaaga | 1290/113 | 1278/494 | 1276/168 |
| ORF70 | - | 20248 | 20788 | 540 | tgcaggccag/***gt***at...***ag***/acccggggagt | 1396/32 | 1117 | 1239 |
| K5/K6 | - | 26454 | 26622 | 168 | ctaaagcagg/***gt***gg...***ag***/acttccacccc | 1518/16 | 1379/92 | 1444/39 |
| **K5/K6** | **-** | **26454** | **26660** | **206** | cgttgatcct/***gt***gc...***ag***/acttccacccc | 1298/11 | 1278/146 | 1354/90 |
| ORF29 | - | 50321 | 53571 | 3250 | caataagaac/***gt***aa...***ag***/agcattcgggg | N/A | 910/19 | 799/9 |
| ORF40-41 | + | 61473 | 61600 | 127 | tggagcctgag/***gt***ga...***ag***/caatggtcgc | 1230/11 | 1259/32 | 1108 |
| **ORF41-50** | **+** | **63368** | **72387** | **9019** | aagatgacaag/***gt***aa...***ag***/ggtaagaagc | N/A | 814 | 936/7 |
| **ORF46-47** | **-** | **68413** | **69389** | **976** | ggcctatact/***gt***ag...***ag***/cggtcaacccc | N/A | 1499 | 1534 |
| ORF50 | + | 71429 | 72387 | 958 | aagatgacaag/***gt***aa...***ag***/ggtaagaagc | 1188/14 | 1109 | 1202/32 |
| K8 | + | 75139 | 75286 | 147 | ctgtagttaag/***gt***ag...***ag*/**gccgaagtat | 1386/187 | 1373/789 | 1382/227 |
| K8 | + | 75379 | 75460 | 81 | agctgcagcag/***gt***at...***ag***/gcattagaag | 1313/167 | 1309/455 | 1303/102 |
| K8/K8.1 | + | 75654 | 76248 | 594 | gactttgtgtg/***gt***aa...***ag***/gatcatattc | 1446/271 | 1442/1061 | 1439/231 |
| K8.1 | + | 75971 | 76248 | 277 | atctccgtcga/***gt***ga...***ag*/**gatcatattc | 1288/61 | 1259/381 | 1289/191 |
| K8.1 | + | 76154 | 76248 | 94 | ccttttcag/***gt***gtat...***ag***/gatcatattc | 1339/313 | 1313/688 | 1337/543 |
| **K8.1** | **+** | **76156** | **76248** | **92** | ccttttcaggt**/*gt***at...***ag***/gatcatattc | 1439/29 | 1385/82 | 1392/55 |
| ORF57 | + | 81934 | 82042 | 108 | catcctagagg/***gt***aa...***ag***/actctgtgtc | 1474/316 | 1478/2782 | 1479/1384 |
| ORF57 | **+** | **82791** | **83362** | **571** | aagcgtactgg/***gt***ga...***ag***/gattgccaaa | 1346/33 | 1371/179 | 1384/62 |
| K10 | - | 88159 | 88260 | 101 | gtgacactag/***gt***at...***ag***/agaacaaagct | 1228/9 | 1012/46 | 1195/42 |
| K10.5 | - | 90662 | 90756 | 94 | gaagaccaag/***gt***aa...***ag***/gccatttgtgg | 1251/7 | 1111 | 1253/41 |
| **Kaposin** | **-** | **114556** | **119100** | **4544** | caggaaccag/***gt***ag...***ag*/**attgaacgcca | N/A | 1046/89 | 1082/37 |
| LANA/vCyclin | - | 124219 | 128138 | 3919 | gtgagtagcg/***cc***cg...***ag***/aagccaccgtc | N/A | 1018/7 | 1099 |
| K14 | + | 129545 | 129693 | 148 | gaggatcctag/***gt***gg...***ag***/gccatggcgg | N/A | 1094/66 | 1097/13 |

The novel splice junctions identified in this study are in bold font. Donor and acceptor sites are in italic, bold font (column 6). Columns 7 and 8 contain the TopHat and HMM generated scores for two replicates at 48 hr (rep1 and rep2) and one at 72 hr post reactivation. These scores reflect the strength of the alignment (HMM splicer) and the number of reads that mapped to the region (TopHat and HMM splicer).
