# Supplementary material for: KSHV 2.0: A Comprehensive Annotation of the Kaposi's Sarcoma-Associated Herpesvirus Genome Using Next-Generation Sequencing Reveals Novel Genomic and Functional Features
Source: PLoS Pathog. 2014 Jan 16;10(1):e1003847. doi: 10.1371/journal.ppat.1003847 (PMC3894221; doi:10.1371/journal.ppat.1003847)
Supplement: Table S5 — mRNA editing in KSHV. mRNA editing events were predicted in silico and were annotated by position and gene. We include in this table a DNA mutation identified in the GQ994935.1 genome in ORF63 at position 102377 (A-to-C change). Three editing events, Kaposin 117809, RTA 72841 and RTA 72795 have been confirmed by Sanger sequencing. (DOCX) [file ppat.1003847.s014.docx]

**Table S5. mRNA editing in KSHV**

| **Position** | **From** | **To** | **Strand** | **Gene** | **Aminoacid change** | **Status** |
| --- | --- | --- | --- | --- | --- | --- |
| 102377 | A | C | + | ORF63 (DNA mutation) | T_445_P | Confirmed |
| 6144 | T | G | + | ORF6 | V_984_G | Not Confirmed |
| 96434 | G | T | - | ORF59 | C_42_F | Not Confirmed |
| 117809 | A | G | - | Kaposin | S_38_Gly | Confirmed |
| 74281 | G | A | + | RTA | A_638_T | Confirmed |
| 72795 | G | A | + | RTA | Silent | Confirmed |
| 17061 | C | A | + | ORF11 polyA cleavage site | Unknown | Not tested |

mRNA editing events predicted in silico by position and gene. The A-to-C change in ORF63 at

position 102377 is a DNA mutation. Three editing events have been confirmed by Sanger sequencing.
