# Supplementary material for: KSHV 2.0: A Comprehensive Annotation of the Kaposi's Sarcoma-Associated Herpesvirus Genome Using Next-Generation Sequencing Reveals Novel Genomic and Functional Features
Source: PLoS Pathog. 2014 Jan 16;10(1):e1003847. doi: 10.1371/journal.ppat.1003847 (PMC3894221; doi:10.1371/journal.ppat.1003847)
Supplement: Table S6 — Alternative start codon usage and internal ORFs in KSHV. From our Ribo-seq data we identified five viral ORFs (ORF70, K6, ORF54, ORF62 and Kaposin) with multiple in frame initiation codons where ribosomes accumulate. The size of the predicted protein products originating from the alternative start codons is indicated in the column labeled amino acids. In addition, five viral ORFs (ORF10, ORF11, K3, ORF20 and K8.1) contain internal initiation codons, in or out of frame with the primary ORF, where we detect initiating ribosomes. The size of the predicted products is indicated in the column labeled amino acids (Nuc.: nucleotides, M: Manual annotation, P: Predicted in silico). (DOCX) [file ppat.1003847.s015.docx]

**Table S6. Alternative start codon usage and internal ORFs in KSHV**

| **Gene** | **Start** | **End** | **Codon** | **Type** | **Prediction** | **Nuc.** | **Aminoacids** |
| --- | --- | --- | --- | --- | --- | --- | --- |
| ORF70 | 21051 | 20038 | ATG | ORF | P | 1013 | 337 |
| ORF70A | 21099 | 20038 | ATG | Alternative start | P | 1061 | 353 |
| ORFK6_vMIP-I | 27374 | 27087 | ATG | ORF | P | 287 | 95 |
| ORFK6A | 27443 | 27087 | CTG | Alternative start | P | 356 | 118 |
| ORFK6B | 27422 | 27087 | ATA | Alternative start | M | 335 | 111 |
| ORF54 | 77483 | 78439 | ATG | ORF | M | 956 | 318 |
| ORF54A | 77552 | 78439 | ATG | Alternative start | M | 887 | 295 |
| ORF62 | 101013 | 100018 | ATG | ORF | P | 995 | 331 |
| ORF62B | 101019 | 100018 | GTG | Alternative start | M | 1001 | 333 |
| ORF62A | 101061 | 100018 | TTG | Alternative start | P | 1043 | 347 |
| Kaposin B | 119121 | 117913 | CTG | ORF | P | 1208 | 402 |
| Kaposin C1 | 119075 | 117738 | CTG | ORF | P | 1337 | 445 |
| Kaposin C2 | 119084 | 117738 | CTG | Alternative start | P | 1346 | 448 |
| ORF10 | 14500 | 15756 | ATG | ORF | M | 1256 | 418 |
| ORF10.2 | 15574 | 15756 | ATG | internal ORF | P | 182 | 60 |
| ORF11 | 15771 | 16994 | ATG | ORF | P | 1223 | 407 |
| ORF11.5 | 15926 | 15991 | ATG | internal ORF | P | 65 | 21 |
| ORFK3 | 19557 | 18589 | ATG | ORF | P | 968 | 322 |
| ORFK3A | 19128 | 18589 | ATG | internal ORF | P | 539 | 179 |
| ORF20 | 35391 | 34429 | ATG | ORF | M | 962 | 320 |
| ORF20A | 35322 | 34429 | CTG | internal ORF | M | 893 | 297 |
| ORF20B | 35202 | 34429 | ATG | internal ORF | P | 773 | 257 |
| k8.1 long | 75732 | 76511 | ATG | ORF | P | 779 | 259 |
| k8.1 short | 75890 | 76511 | ATG | internal ORF | P | 622 | 206 |

Five viral ORFs (ORF70, K6, ORF54, ORF62 and Kaposin) have multiple in frame initiation codons. The size of the resulting protein products is indicated in the column labeled aminoacids. Five viral ORFs (ORF10, ORF11, K3, ORF20 and K8.1) contain internal initiation codons in or out of frame with the primary ORF. The size of the product of the internal ORF is indicated in the column labeled aminoacids (M: Manual annotation, P: Predicted in silico)
