# Supplementary material for: KSHV 2.0: A Comprehensive Annotation of the Kaposi's Sarcoma-Associated Herpesvirus Genome Using Next-Generation Sequencing Reveals Novel Genomic and Functional Features
Source: PLoS Pathog. 2014 Jan 16;10(1):e1003847. doi: 10.1371/journal.ppat.1003847 (PMC3894221; doi:10.1371/journal.ppat.1003847)
Supplement: Table S7 — Upstream and small ORFs are widely distributed in KSHV. The translation boundaries for uORFs and sORFs were predicted by the SVM based on the Ribo-seq data from harringtonine treated cells at 72 h post reactivation. The ORF coordinates were then manually curated. The translation efficiency (TE) for the ORFs larger than 15 amino acids was determined as Footprint-rpkM/mRNA-rpkM. For the calculation we excluded the first 45 nucleotides from the start codon. (M: Manual annotation, P: Predicted in silico, N/A: not available, *: Start codons are ambiguous). (DOCX) [file ppat.1003847.s016.docx]

**Table S7. Upstream and small ORFs are widely distributed in KSHV**

| **Gene** | **Start** | **Stop** | **Strand** | **Codon** | **Type** | **Size** | **Prediction** | **TE** |
| --- | --- | --- | --- | --- | --- | --- | --- | --- |
| ORFK4.1a | 22517 | 22416 | - | ATG | sORF | 33 | P | 6.98 |
| ORFK4.1d | 22610 | 22545 | - | CTG | sORF | 21 | P | 5.78 |
| ORFK4.1e | 22653 | 22627 | - | ATG | sORF | 8 | P | N/A |
| ORFK4.1c | 22806 | 22723 | - | CTG | sORF | 27 | P | 7.30 |
| ORFK4.1b | 22850 | 22545 | - | ATG | sORF | 101 | P | 7.43 |
| 1.4KbB | 24871 | 24915 | + | ATG | sORF | 14 | P | N/A |
| 1.4KbC | 24921 | 25058 | + | ATG | sORF | 45 | M | 0.43 |
| PAN 1.1 | 28655 | 28768 | + | ATG | sORF | 37 | P | 0.14 |
| PAN 1.2 | 28831 | 28965 | + | CTG | sORF | 44 | P | 0.07 |
| PAN 1.3 | 28888 | 28965 | + | ATG | sORF | 25 | P | 0.02 |
| 43.1-AS | 63214 | 63228 | + | ATG | sORF | 4 | P | N/A |
| ORF43.2-AS | 63254 | 63295 | + | ATG | sORF | 13 | M | N/A |
| ORF50AS* | 74130 | 74222 | - | CTG | sORF | 30 | P | 3.94 |
| K15.1 | 135846 | 135938 | - | CTG | sORF | 30 | P | 10.17 |
| ORF6.1 | 3027 | 3203 | + | ATG | uORF | 58 | P | 6.78 |
| ORF6.2 | 3150 | 3203 | + | ATT | uORF | 17 | P | 19.37 |
| ORF10.1 | 14451 | 14531 | + | CTG | uORF | 26 | P | 6.82 |
| ORF11.1 | 15633 | 15722 | + | CTG | uORF | 29 | P | 10.18 |
| ORF11.2 | 15648 | 15722 | + | CTG | uORF | 24 | P | 13.78 |
| ORF11.3 | 15693 | 15722 | + | CTG | uORF | 9 | P | N/A |
| ORF11.4 | 15745 | 15756 | + | ATG | uORF | 3 | P | N/A |
| vIL6.6 | 17873 | 17862 | - | ATG | uORF | 3 | M | N/A |
| vIL6.5 | 17915 | 17877 | - | GTG | uORF | 14 | P | N/A |
| vIL6.4 | 18003 | 17902 | - | ATG | uORF | 33 | P | 7.23 |
| vIL6.3 | 18047 | 17985 | - | CTG | uORF | 20 | P | 21.34 |
| vIL6.2 | 18086 | 18057 | - | ATG | uORF | 9 | P | N/A |
| vIL6.1 | 18116 | 18057 | - | ATG | uORF | 19 | P | 9.70 |
| ORFK4A | 21820 | 21743 | - | ATC | uORF | 25 | P | 7.11 |
| ORFK5.1 | 26569 | 26555 | - | ATG | uORF | 4 | P | N/A |
| ORFK6.1 | 27647 | 27615 | - | ATG | uORF | 10 | P | N/A |
| ORF21.1 | 35151 | 35177 | + | CTG | uORF | 8 | M | N/A |
| ORF25.1* | 42345 | 42380 | + | ATG | uORF | 11 | P | N/A |
| ORF28.1 | 48758 | 48811 | + | GTG | uORF | 17 | M | 19.65 |
| ORF30.1 | 50317 | 50358 | + | ATG | uORF | 13 | P | N/A |
| ORF34.1 | 54399 | 54485 | + | ATG | uORF | 28 | P | 3.01 |
| ORF35.1 | 55419 | 55445 | + | ATG | uORF | 8 | M | N/A |
| uORF35.2 | 55442 | 55474 | + | ATG | uORF | 10 | P | N/A |
| ORF37.1 | 57040 | 57126 | + | ATG | uORF | 28 | P | 3.12 |
| ORF38.1 | 58251 | 58259 | + | TTG | uORF | 2 | P | N/A |
| ORF38.2 | 58455 | 58589 | + | CTG | uORF | 44 | P | 5.93 |
| ORF45.1 | 68447 | 68364 | - | ATA | uORF | 27 | P | 10.91 |
| ORF49.1 | 72425 | 72384 | - | ACG | uORF | 13 | M | N/A |
| ORF55.1 | 79501 | 79340 | - | ATG | uORF | 53 | P | 2.78 |
| ORF61.2 | 100071 | 100018 | - | CTG | uORF | 17 | P | 15.96 |
| ORF61.1 | 100095 | 100018 | - | GTG | uORF | 25 | P | 12.13 |
| ORF65.1 | 112321 | 112289 | - | CTG | uORF | 30 | P | 7.76 |
| ORF69.1 | 116043 | 116138 | + | ATG | uORF | 31 | P | 6.80 |
| ORF72.1 | 124182 | 124108 | - | ATG | uORF | 24 | P | 2.47 |
| ORF75.1 | 134809 | 134729 | - | CTG | uORF | 26 | P | 33.79 |
| ORF75.2 | 134894 | 134817 | - | CTG | uORF | 25 | P | 11.19 |

The translation boundaries for uORFs and sORFs annotated were predicted by the SVM based on the Ribo-seq data from harringtonine treated cells at 72h post reactivation. The ORF coordinates were then manually curated. The translation efficiency (TE) was determined as Footprint-rpkM/mRNA-rpkM. For the calculation we excluded the first 45 nucleotides from the start codon. M: Manual annotation, P: Predicted in silico, N/A: not available, *: Start codons are ambiguous
